# Dual engagement of Spike and ACE2 by annexin A5 contributes to pleiotropic SARS-CoV-2 inhibition

**DOI:** 10.1038/s41598-026-58451-9

**Published:** 2026-06-20

**Authors:** Arundhasa Chandrabalan, Qi-Tong Lin, Khandaker Atkia Fariha, Paul Solis-Reyes, Connor G. Richer, Ryan M. Troyer, Stephen D. Barr, Qingping Feng, Peter B. Stathopulos

**Affiliations:** 1 Department of Physiology and Pharmacology, Schulich School of Medicine and Dentistry, University of Western Ontario, London, ON N6A 5C1 Canada; 2 Department of Microbiology & Immunology, University of Western Ontario, London, ON N6A 5C1 Canada; 3 Present Address: Biological Sciences, Sunnybrook Research Institute, Toronto, ON Canada

**Keywords:** COVID-19, SARS-CoV-2, Spike-RBD, ACE2, Annexin A5, Diseases, Drug discovery, Medical research, Microbiology

## Abstract

COVID-19 is primarily a respiratory tract infection caused by severe acute respiratory syndrome coronavirus 2 (SARS-CoV-2), which can enter host airway epithelial cells through angiotensin-converting enzyme 2 (ACE2) receptors. Emerging SARS-CoV-2 variants are a roadblock to irradicating the disease. Given recombinant human annexin A5 (Anx5) inhibits proinflammatory responses, improves survival in sepsis models and binds to several receptors and lipids, we hypothesized that Anx5 impedes SARS-CoV-2 viral entry and lessens disease severity. To assess SARS-CoV-2 Spike-receptor binding domain (RBD) and ACE2 interactions with Anx5, the recombinant proteins were expressed and isolated, and interactions were evaluated using solution NMR, microscale thermophoresis, steady-state fluorescence and size-exclusion chromatography coupled to multi-angle light scattering. Remarkably, we found that Anx5 binds to both the Spike-RBD and ACE2. Further, Anx5 induced extensive ^15^N-Spike-RBD amide resonance broadening, most concentrated in the region that engages with ACE2 and least enriched on the opposite face, highlighting the ACE2 interface as the primary site of Anx5 binding. Finally, we demonstrate that Anx5 inhibits SARS-CoV-2 pseudoviral entry and reduces viral burden while enhancing survival in SARS-CoV-2-infected mammalian cells. Collectively, our findings demonstrate that Anx5 directly engages both Spike-RBD and ACE2, inhibits SARS-CoV-2 pseudoviral entry and reduces viral burden while enhancing survival of infected mammalian cells, supporting further investigation of Anx5 as a multi-functional anti-SARS-CoV-2 therapeutic.

**Supplementary Information:**

The online version contains supplementary material available at https://doi.org/10.1038/s41598-026-58451-9.

## Introduction

Coronavirus disease 2019 (COVID-19) is primarily a respiratory tract infection caused by severe acute respiratory syndrome coronavirus 2 (SARS-CoV-2). While vaccines have been effective in reducing the number of COVID-19 cases, the disease is not likely to be eradicated due to the emergence of SARS-CoV-2 variants that evade immunity to currently available vaccines or previous infections^1^. It is therefore essential to develop new and effective therapies for COVID-19. SARS-CoV-2 enters the host cells predominantly via binding to the angiotensin-converting enzyme 2 (ACE2) receptor^2^, although alternative receptors supporting SARS-CoV-2 entry have also been discovered (e.g., Neuropilin-1, CD209/DC-SIGN and CD209L/L-SIGN, AXL, KREMEN1 and ASGR1^3–6^). SARS-CoV-2 initiates infection by binding to the ACE2 extracellular domain (i.e., residues S19-D615) via the receptor binding domain (i.e., RBD, residues R319-F541) located within the S1 subunit of the glycosylated homotrimeric Spike protein. Following this initial attachment, viral entry necessitates membrane fusion, a process triggered by the subsequent cleavage of the Spike protein at the S2’ site by host cell surface proteases such as transmembrane protease serine 2 (TMPRSS2)^7–10^. Heparan sulfate on the cell surface has also been shown to assist in SARS-CoV-2 cell entry^11^. Importantly, binding of isolated Spike-RBD to ACE2 occurs independent of proteolytic cleavage in vitro^9^ with a high binding affinity (i.e., equilibrium dissociation constant, K_D_ = ~ 5-150 nM)^9,10,12–16^.

Once inside the host cell, the virus replicates rapidly and causes a systemic pro-inflammatory response with increased cytokine levels [e.g., interleukin-6 (IL-6), IL-10, and tumor necrosis factor-α (TNF-α)], leukocytosis, lymphopenia in CD4^+^ and CD8^+^ T cells, and decreased interferon-α (IFN-α) expression in CD4^+^ T cells. Additionally, thrombocytopenia, elevated prothrombin time and high levels of D-dimers suggest coagulopathy in COVID-19^17^. Though the initial target organ is the lung causing pneumonia, acute respiratory distress syndrome (ARDS) and acute pulmonary embolism, the thrombo-inflammatory process may consecutively affect all organs of the body^18^. The imbalance between pro- and anti-inflammatory responses causes dysfunction of multiple organs including the lung, liver, kidney and cardiovascular system as well as coagulopathy, leading to septic shock and high mortality^19^. Sepsis develops when the initial host response to infection becomes amplified, leading to an exaggerated systemic pro-inflammatory response. Notably, sepsis develops in all severe cases ~ 1–2 days before ARDS, and acute pulmonary embolus occurs in ~ 30% of these patients, suggesting that sepsis and the thrombo-inflammatory response are key contributors to the development of ARDS and multi-organ failure^18,20^. Thus, an effective treatment of sepsis and thrombo-inflammation has the potential to prevent ARDS and multi-organ dysfunction in COVID-19. Previously, we and others have established that recombinant human annexin A5 (Anx5) inhibits pro-inflammatory responses and improves survival in rodent models of bacterial sepsis^21–23^.

Anx5 is an endogenous cytosolic protein broadly expressed in tissues and cells including the placenta, kidney, heart, lungs and highly in vascular endothelial cells with potent anti-apoptotic, anti-inflammatory, and anti-coagulant properties^24–28^. Structurally, Anx5 is composed of four α-helical domains arranged to form a curved shape with distinct concave and convex faces^29^. Anx5 binds with high affinity to negatively charged phosphatidylserine in a calcium (Ca^2+^)-dependent manner, interacts with receptors and proteins through leucine-rich repeats, and can inhibit the function of various cell-surface receptors^22,23,30,31^. Beyond these host-protective roles, members of the annexin family have been increasingly implicated in host-pathogen interactions, serving as either co-receptors or entry inhibitors for various enveloped viruses^32,33^. The Ca^2+^ and phosphatidylserine binding sites are located on the membrane-facing convex side of Anx5^29^. Notably, Anx5 self-assembles as trimers and has a unique ability to form a two-dimensional lattice on membrane lipid bilayers, acting as a shield that limits interaction with extracellular factors^34^.

The use of Anx5 as a treatment for COVID-19 and sepsis has been studied in clinical trials. A single-centre, randomized Phase II trial demonstrated feasibility and safety of administering human recombinant Anx5 (SY-005) in patients with severe COVID-19^35,36^. Anx5 safety was further confirmed in a recent multicenter, randomized Phase IIa trial in patients with sepsis (ClinicalTrials.gov Identifier: NCT04898322). However, additional studies are needed to establish therapeutic efficacy in both COVID-19 and sepsis.

Building on these known roles in receptor modulation and viral entry interference, we hypothesized that Anx5 can inhibit SARS-CoV-2-Spike interactions with ACE2, thereby limiting viral infection and disease severity. To test this hypothesis, we expressed and isolated recombinant Anx5, ACE2 and SARS-CoV-2 Spike-RBD and evaluated the protein-protein interactions in vitro as well as infection and survival in cellulo. Binding affinities were quantified using solution NMR spectroscopy, steady-state fluorescence and microscale thermophoresis (MST), while protein-protein complex formation was evaluated by size-exclusion chromatography coupled with multi-angle light scattering (SEC-MALS). Strikingly, Anx5 was found to bind both Spike-RBD and ACE2, and solution NMR revealed that the Anx5 binding site on Spike-RBD overlaps with the ACE2 interface, suggesting competitive binding. Functionally, treatment with recombinant human Anx5 inhibited pseudoviral entry and enhanced survival in SARS-CoV-2-infected mammalian cells. Collectively, our findings indicate that Anx5 blocks SARS-CoV-2 infection through dual mechanisms and highlight its potential as a multi-target antiviral as well as post-infection therapeutic.

## Results

### SARS-CoV-2 Spike-RBD, human ACE2 and Anx5 can be efficiently expressed and purified from *E. coli*

**Fig. 1:**
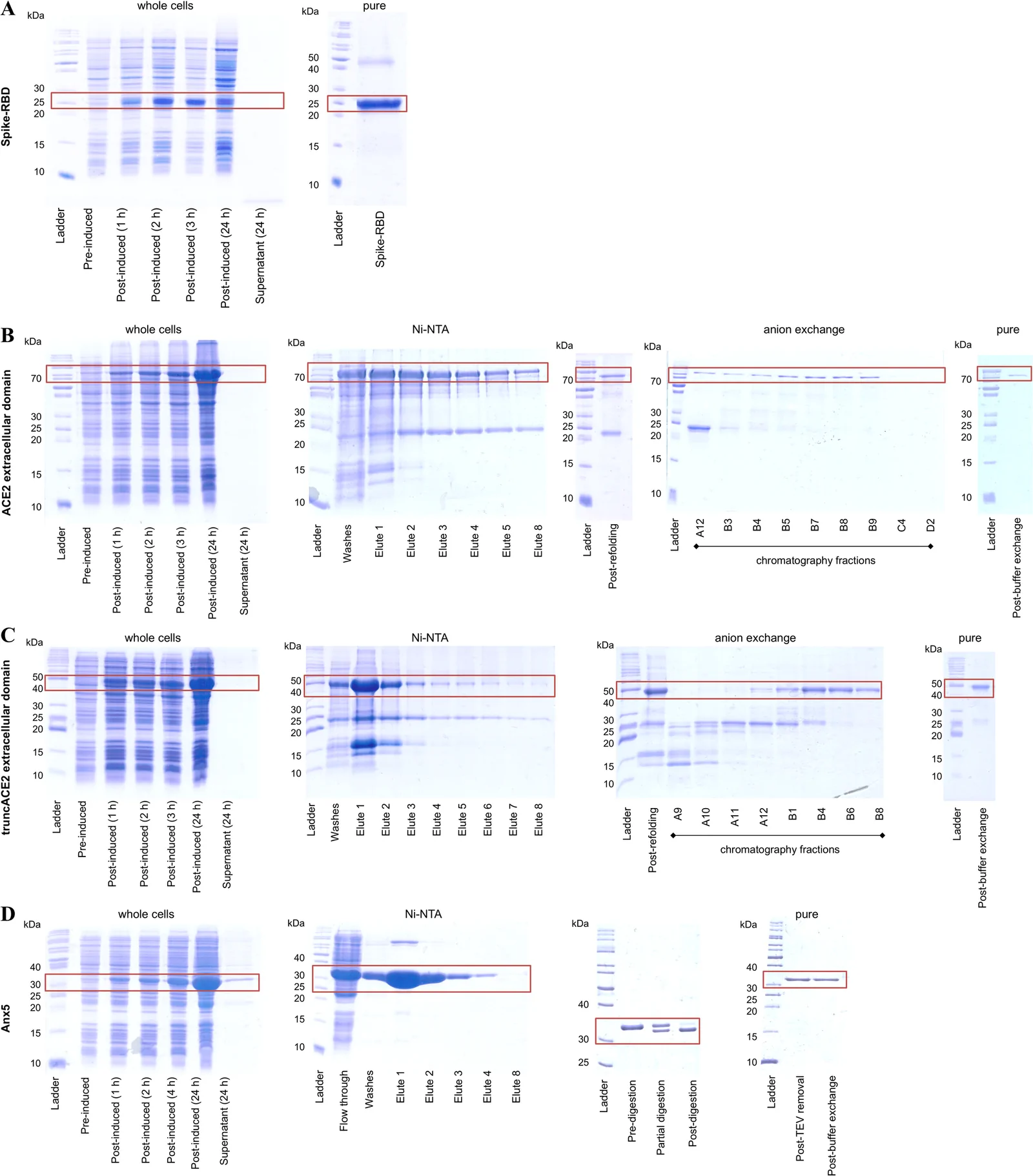
Expression and purification of recombinant proteins. (**A**) SARS-CoV-2 Spike-RBD expression in *E. coli.* The time-dependency of expression and final 6×His-Spike-RBD (27.42 kDa) purity is highlighted. (**B**) Full-length ACE2 extracellular domain expression in *E. coli*. The time-dependency of expression and purity of 6×His-ACE2 (71.41 kDa) after the Ni-NTA affinity, refolding and anion-exchange chromatography steps are highlighted. (**C**) Truncated ACE2 extracellular domain expression in *E. coli*. The time-dependency of expression and purity of 6×His-truncACE2 (46.14 kDa) after the Ni-NTA affinity, refolding and anion-exchange chromatography are highlighted. (**D**) Anx5 expression in *E. coli*. The time-dependency of expression and purity after Ni-NTA affinity chromatography and TEV-mediated His-tag of Anx5 (35.81 kDa) are shown. In *A-D*, 15% (w/v) SDS-PAGE gels were run in Tris-Glycine buffer and stained with Coomassie-blue. Red boxes highlight the location of the recombinant proteins of interest.

To assess the SARS-CoV-2 Spike-RBD and ACE2 interactions with Anx5, the cDNA region encoding Spike-RBD (R319-F541), full-length ACE2 (S19-D615), and truncated ACE2 (truncACE2; S19-E398) were separately cloned into a pET28a vector, and Anx5 (M1-D320) into a pEX-N-His vector for high level protein expression. Because full-length ACE2 was prone to degradation and could not be concentrated to the levels required for NMR experiments, we also generated truncACE2, which retains the α-helix and Lys353-containing distal loop that form the principal Spike-RBD binding interface in ACE2 subdomain I and has been utilized in similar interaction studies^14,37^. Using these constructs, all proteins were successfully expressed in *E. coli* (Fig. 1). 6×His-Spike-RBD (theoretical pI = 8.75, mass = 27.42 kDa, Fig. 1A), 6×His-ACE2 (theoretical pI = 5.49, mass = 71.41 kDa, Fig. 1B), and 6×His-truncACE2 (theoretical pI 5.29, mass = 46.14 kDa, Fig. 1C) recombinant proteins were extracted efficiently under denaturing conditions, whereas Anx5 (theoretical pI 5.04, mass = 35.81 kDa, Fig. 1D) was extracted under non-denaturing conditions, at pH 7.4. The crude proteins were purified by Ni-NTA metal affinity chromatography and dialysis (Fig. 1A–D) with only full-length and truncated ACE2 proteins requiring further purification through anion-exchange chromatography (Fig. 1B and C). Using these approaches, the recombinant proteins were produced with the following yields per 1 L of LB culture: 12 mg Spike-RBD, 0.4 mg ACE2, 0.8 mg truncACE2, and 68 mg Anx5. Since our Spike-RBD showed aberrantly rapid migration on SDS-PAGE gels compared to the theoretical molecular weight, the identity of this recombinant protein was further confirmed by electrospray ionization mass spectrometry. The mass spectrometry revealed a molecular weight of 27412.6 Da, consistent with the intact protein containing four intramolecular disulfide bonds (Fig. S1).

### Recombinant Spike-RBD adopts a mixture of β-sheet, α-helix, and random coil

**Fig. 2:**
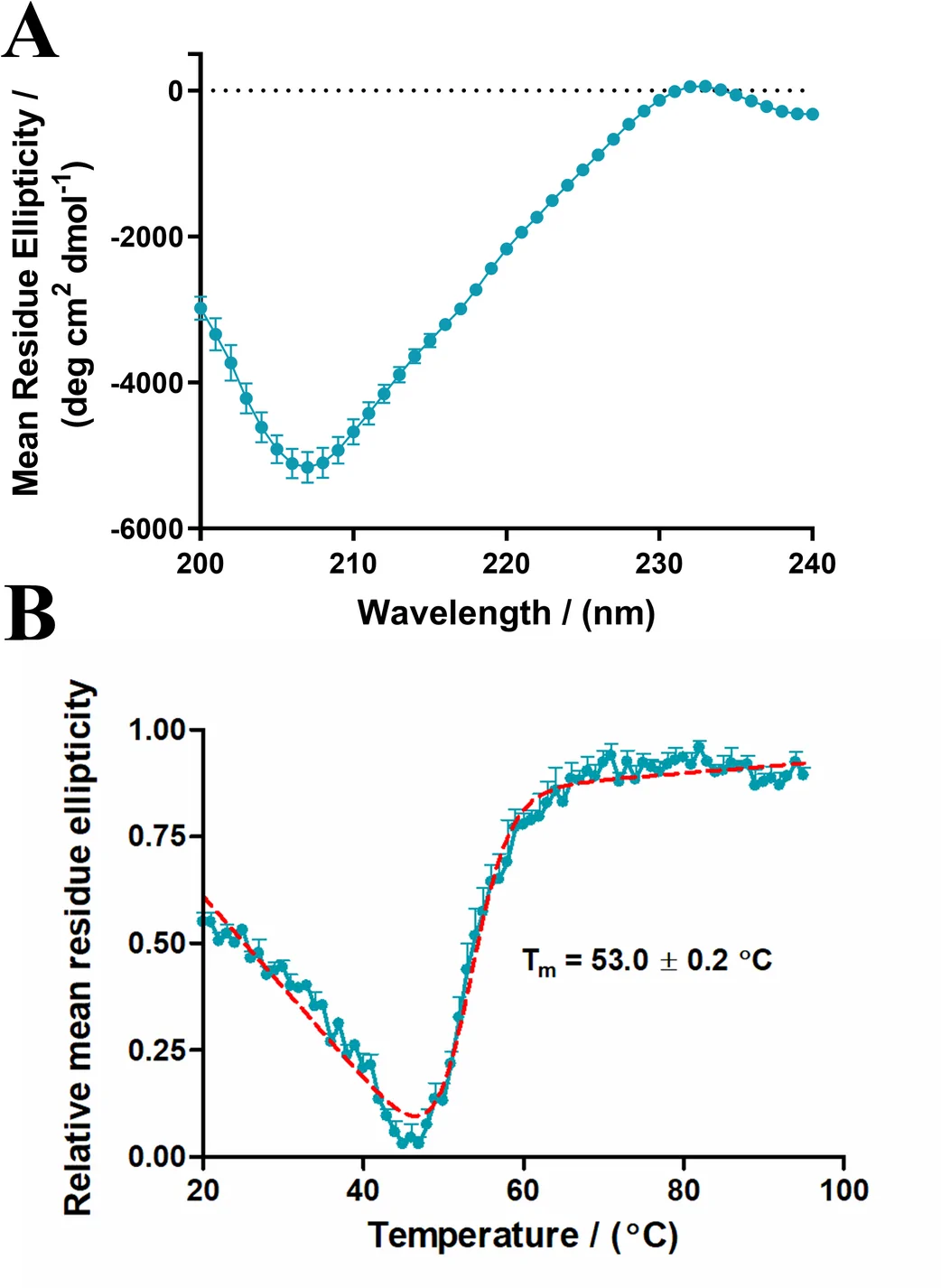
Secondary structure and thermal stability of Spike-RBD. (**A**) Far-UV CD spectrum of Spike-RBD at 20 °C. (**B**) Thermal melt curve of Spike-RBD acquired at 222 nm. The dashed red line is the Boltzmann sigmoidal with sloping baseline fit to the data to extract the midpoint of temperature denaturation (T_m_). The data are presented as means ± SEM of *n* = 3 (spectra) and *n* = 4 (melts) experiments. In *A and B*, data were acquired with 0.4 mg/mL protein in 20 mM Na_2_HPO_4_ (pH = 7.4) buffer.

Given that Spike-RBD contains nine Cys residues that necessitated the presence of oxidized (GSSG) and reduced glutathione (GSH) in the refolding buffer, we next assessed whether the domain was correctly folded. First, we determined the secondary structure of Spike-RBD by far-UV circular dichroism (CD) spectroscopy (Fig. 2A). A strong ellipticity minimum was observed at ~ 208 nm that gradually decayed to baseline by ~ 230 nm, with clear signal at ~ 215 and ~ 222 nm, indicative of β-sheet, α-helix, and random coil. These signals are fully consistent with the known structure of this domain^38,39^. To further confirm the integrity of the fold, we also assessed thermal stability of the domain by monitoring changes in ellipticity as a function of increasing temperature at 222 nm. The thermal melting profile exhibited an initial gain in negative ellipticity at 222 nm followed by an abrupt unfolding transition. A Boltzmann sigmoidal fit to the data revealed an apparent midpoint of temperature denaturation (T_m_) of 53.0 ± 0.2 °C (Fig. 2B). Thus, Spike-RBD spontaneously adopts the expected secondary structure and undergoes cooperative unfolding, despite the high Cys content. Note that the correct/expected fold was also confirmed by solution NMR spectroscopy (see below).

### Anx5 interacts with both Spike-RBD and ACE2

**Fig. 3:**
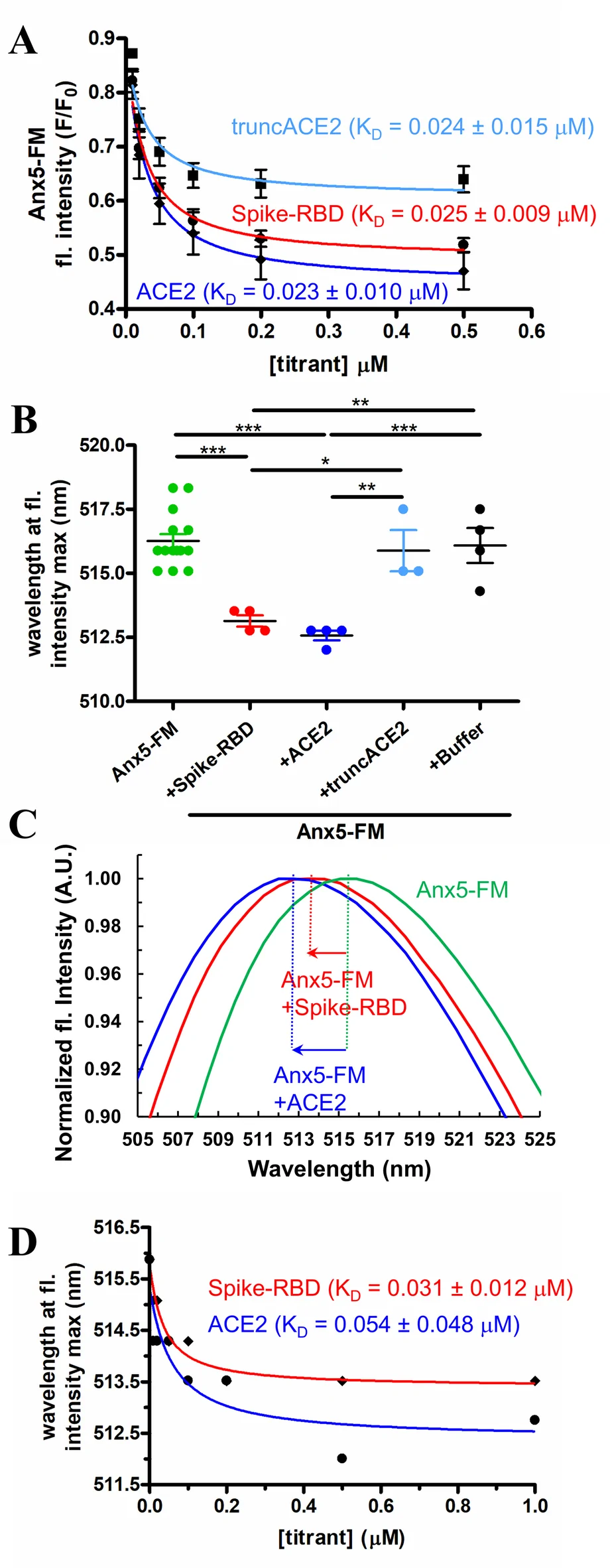
Steady-state fluorescence evaluation of Anx5 interactions with Spike-RBD and ACE2. (**A**) Change in Anx5-fluorescein fluorescence as a function of increasing Spike-RBD (red), ACE2 (blue) and truncACE2 (cyan) concentration. (**B**) Comparison of the wavelengths at the maximum fluorescence intensity (λ_max_) for Anx5-FM in the absence (green) and presence of 0.5 µM Spike-RBD (red), ACE2 (blue), truncACE2 (light blue) and buffer (black). (**C**) Representative fluorescence emission peaks of Anx5-FM in the absence (green) and presence of 0.5 µM Spike-RBD (green) and ACE2 (blue). The vertical dotted lines highlight the peak maximum for each of the spectra and the horizontal arrows show the shift to lower λ_max_ upon binding Spike-RBD (red) and ACE2 (blue). (**D**) Representative binding isotherms constructed from plots of λ_max_ versus increasing Spike-RBD and ACE2 concentration. In *A-D*, Anx5-fluorescein was at 10 nM, experiments were performed in 20 mM Na_2_HPO_4_ (pH = 7.4) buffer at 22.5 °C. In *A* and *D*, K_D_ values were extracted from the data using one-site binding model fits (solid lines) taking into account protein concentration. In *A* and *B*, data are means ± SEM of *n* = 3–4 experiments, except for free Anx5-FM λ_max_ (in *B*), where *n* = 15. In *B*, statistical analysis was One-way ANOVA followed by Bonferroni post-hoc test, where ***p < 0.001, **p < 0.01, *p < 0.05.

With the recombinant proteins in hand, we next evaluated binding of human Anx5 to Spike-RBD and human ACE2. First, we labeled Anx5 with fluorescein at free Cys residues and evaluated steady-state fluorescence. We observed decreases in Anx5-fluorescein (Anx5-FM) fluorescence with increasing Spike-RBD concentrations; moreover, fitting the resultant curve to a one-site binding model revealed a K_D_ = 0.025 ± 0.01 µM (Fig. 3A). Titrating ACE2 or truncACE2 into Anx5-FM also decreased fluorescence and revealed similar fitted K_D_ values of 0.023 and 0.024 µM, respectively (Fig. 3A), demonstrating comparable Anx5 binding behavior between the intact and truncated forms. However, control titrations of buffer into Anx5-FM solution also caused decreases in fluorescence intensity, albeit to a lesser extent than titrations with proteins (see Methods). Thus, we also evaluated the wavelengths at maximum fluorescence intensity (λ_max_). Changes in λ_max_ occur due to stoichiometry and/or folding changes and are independent of intensity changes. Whereas control buffer titrations caused no significant changes in λ_max_, both Spike-RBD and ACE2 caused a significant blue shift in the Anx5-FM λ_max_, confirming binding (Fig. 3B and C). Fitting the change in λ_max_ versus Spike-RBD and ACE2 concentration revealed K_D_ values of 0.031 ± 0.012 µM and 0.054 ± 0.048 µM, respectively (Fig. 3D and Table S1), reinforcing the apparent binding we observed with the intensity changes.

**Fig. 4:**
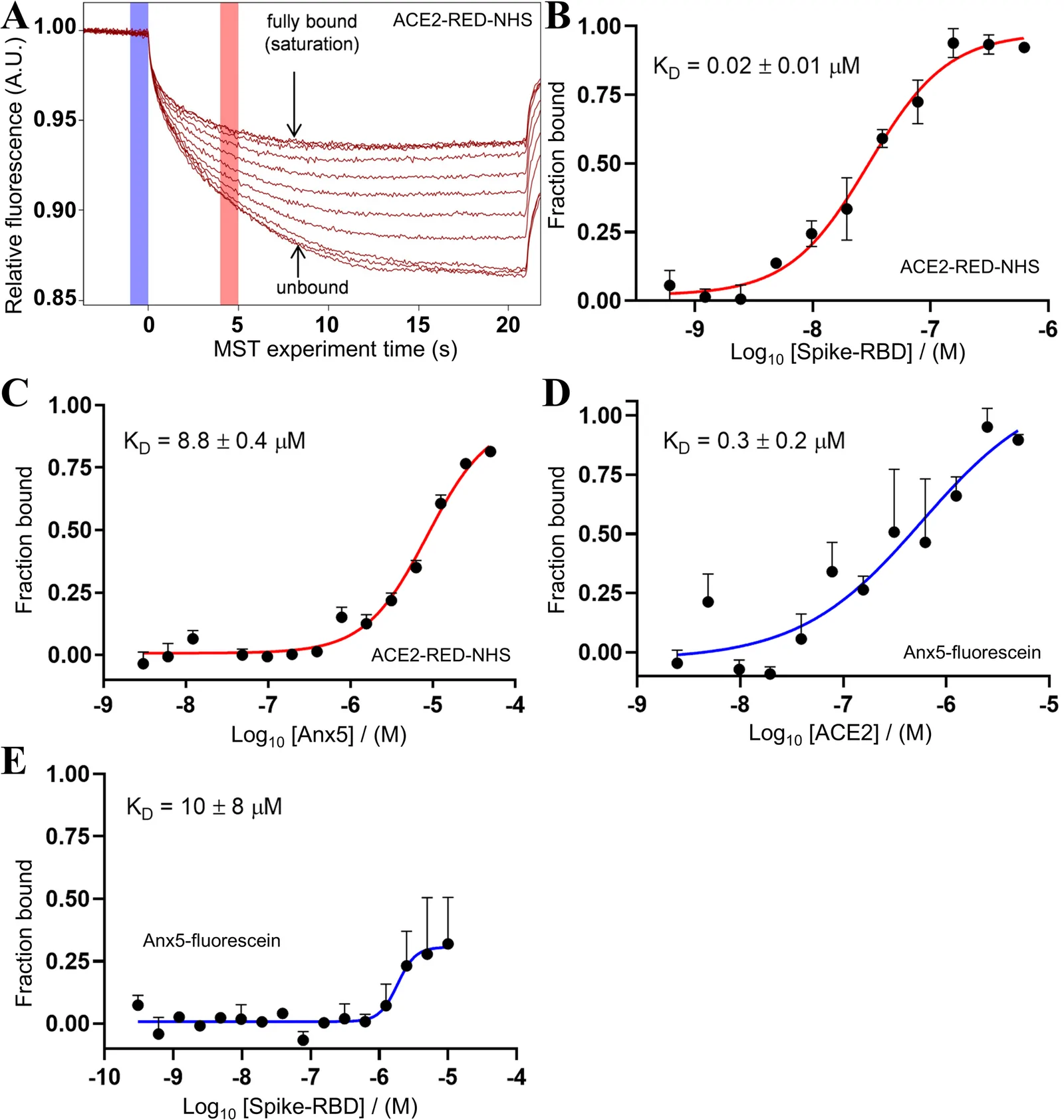
MST analysis of Anx5 interactions with Spike-RBD and ACE2. (**A**) Representative ACE2-RED-NHS MST traces as a function of Spike-RBD over a 10-step two-fold dilution series (0.0006–0.6 µM, bottom – top, respectively). (**B**) MST-derived fractional saturation curve of Spike-RBD binding to ACE2-RED-NHS. (**C**) MST-derived fractional saturation curve of Anx5 binding (0.003–50 µM) to ACE2-RED-NHS. (**D**) MST-derived fractional saturation curve of ACE2 binding (0.002–5 µM) to Anx5-fluorescein. (**E**) MST-derived fractional saturation curve of Spike-RBD binding (0.0003–10 µM) to Anx5-fluorescein. In *A-E*, data were acquired with 20 nM of the labeled protein and experiments were performed in 20 mM Na_2_HPO_4_ (pH = 7.4), 0.05% (v/v) tween-20 at 22 °C. K_D_ values were extracted from the data using one-site binding model fits (solid lines) taking into account protein concentration. Data are means ± SEM of *n* = 3–4 separate experiments.

Since the steady-state fluorescence experiments were performed with low ionic strength, ideal for solution NMR spectroscopy, and Anx5-FM tended to stick to experimental materials (see Methods), we next re-evaluated the binding using MST in the presence of 0.05% (v/v) Tween-20. Detergent is routinely added to MST experiments to prevent protein adherence to capillaries and protein aggregation. In these MST experiments we separately used Anx5-FM and ACE2 labeled with the primary amine-reactive RED-NHS (i.e. ACE2-RED-NHS). As a positive control, we first assessed Spike-RBD interactions with ACE2-RED-NHS. As expected, the temperature-induced fluorescence and thermophoretic changes measured by MST demonstrated a tight interaction between Spike-RBD and ACE2-RED-NHS, with an extracted K_D_ = 0.02 ± 0.01 µM (Fig. 4A and B), within the previously reported range of 0.005–0.150 µM^9,10,12–16^. Consistent with our steady-state fluorescence measurements, Anx5 also showed an interaction with ACE2-RED-NHS by MST, with a K_D_ = 8.8 ± 0.4 µM (Fig. 4C). We additionally performed the reverse MST experiment, titrating unlabeled ACE2 into Anx5-FM and finding a K_D_ = 0.3 ± 0.2 µM (Fig. 4D). Finally, a titration of unlabeled Spike-RBD with Anx5-FM further confirmed the Spike-RBD: Anx5-FM interaction, revealing a K_D_ = 10 ± 8 µM (Fig. 4E). Collectively, our steady-state and MST experiments indicate that Anx5 binds to both Spike-RBD and ACE2 with ~ µM - nM affinity (Table S1).

### Anx5 interactions with Spike-RBD and ACE2 are in fast exchange

Having determined Anx5 interacts with both Spike-RBD and ACE2, we next evaluated the stoichiometry of the proteins alone and in combination to reveal information about the exchange dynamics using SEC-MALS. Spike-RBD (3 mg/ml, theoretical MW: 27.42 kDa) eluted as a single broad symmetrical peak and with a MALS-determined MW of 28.7 ± 0.5 kDa (Fig. S2A). Anx5 (1 mg/ml, theoretical MW: 35.81 kDa) also eluted as a single symmetrical peak with a measured MW of 30.4 ± 0.2 kDa (Fig. S2A). Thus, both Spike-RBD and Anx5 exist primarily as monomers under the dilute SEC-MALS conditions. Interestingly, injection of a mixed Spike-RBD (3 mg/ml) and Anx5 (1 mg/ml) sample did not appreciably alter the elution profile of either protein, with Anx5 revealing a SEC-MALS determined MW of 35.0 ± 0.3 kDa and Spike-RBD MW of 22.9 ± 0.2 kDa (Fig. S2A). Coomassie blue-stained SDS-PAGE gels of the individual and mixed elution fractions confirmed the proteins were intact but showed no co-elution at lower/complexed volumes (Fig. S2B–D).

In contrast to monomeric Anx5 and Spike-RBD, ACE2 (0.7 mg/ml, theoretical MW: 71.41 kDa) showed a broad polydisperse elution profile with multiple peaks, corresponding to monomer, dimer and oligomer (Fig. S2E). Co-injection of ACE2 (0.7 mg/ml) with Anx5 (1 mg/ml) diminished absorbance of ACE2-related peaks, likely due to precipitation of complexed protein. Nevertheless, the elution profile of the mixed sample was similar to the combined profiles of the individually injected samples, and no complexed co-elution at earlier volumes was evident on Coomassie blue-stained SDS-PAGE gels (Fig. S2F–H).

Since we were only able to concentrate ACE2 to a maximum of ~ 0.7-1 mg/mL in our Na_2_HPO_4_ buffer, we also tried SEC-MALS experiments using truncACE2 (1.5 mg/ml, theoretical MW: 46.14 kDa). TruncACE2 eluted primarily as two partially overlapping symmetrical peaks with SEC-MALS-determined MWs of 97 ± 2 and 51 ± 6 kDa, corresponding to dimer and monomer, respectively (Fig. S2I). Co-injection of truncACE2 (1.5 mg/ml) with Anx5 (1 mg/ml) showed small shifts toward lower retention volumes [i.e. elution V_max_ = 11.68, 12.55 (truncACE2 dimer), 14.03 (truncACE2 monomer), 14.86 ml (Anx5)] compared to the individual injections [i.e. elution V_max_ = 11.92, 12.63 (truncACE2 dimer), 14.09 (truncACE2 monomer), 14.91 ml (Anx5)]; further, the truncACE2 monomer peak showed lower relative intensity in the mixed compared to the individual injection (Fig. S2I). However, Coomassie blue-stained SDS-PAGE analysis of the elution fractions showed no evidence of earlier co-elution (Fig. S2J–L).

Together, the SEC-MALS experiments indicate that in isolation and under dilute conditions Anx5 and Spike-RBD proteins exist primarily as monomers, whereas ACE2 and truncACE2 form dimers and higher order oligomers. Further, Anx5:ACE2, Anx5:truncACE2 and Anx5:Spike-RBD interactions do not exhibit co-elution, suggesting fast exchange dynamics associated with the ~ 20-30-fold SEC column dilution.

### Anx5 interacts in the region of Spike-RBD that engages ACE2

**Fig. 5:**
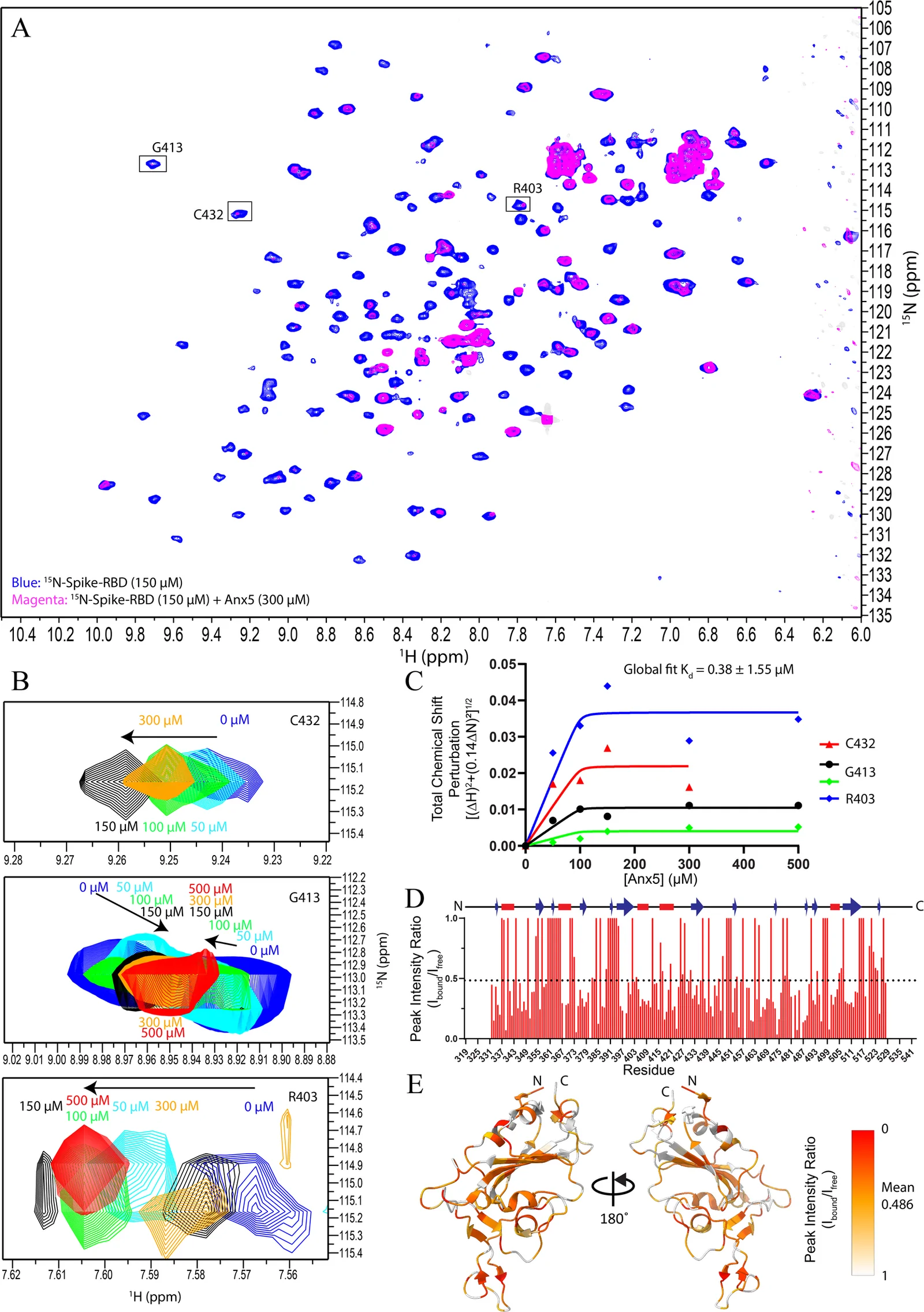
Solution NMR analysis of ^15^N-Spike-RBD interactions with Anx5. (**A**) Overlaid ^1^H-^15^N HSQC NMR spectra of free (apo) ^15^N-Spike-RBD (~ 150 µM, blue) and ^15^N-Spike-RBD (~ 150 µM) in complex with Anx5 (~ 300 µM, magenta). (**B**) Zoomed views of representative ^15^N-Spike-RBD amide crosspeaks undergoing fast exchange CSPs as a function of increasing Anx5 concentration. (**C**) Simultaneous fits of ^15^N-Spike-RBD amide crosspeak CSPs to a one-site binding model taking into account protein concentration, extracting a global K_D_ = 0.38 ± 1.6 µM. (**D**) Intensity ratio of bound (I)/free (I_0_) ^15^N-Spike-RBD amide crosspeaks as a function of residue number. Spike-RBD secondary structure components from 6LZG.pdb^9^ is shown at top. Dashed line shows the mean I/I_0_ ratio across all residues. (**E**) Gradient plot of the residue-specific I/I_0_ ratios on the 3D structure of the Spike-RBD (6LZG.pdb^9^). The three-colour gradient is from minimum (I/I_0_ ~1, white) to mean (I/I_0_ ~0.5, orange) to maximum change (I/I_0_ ~0, red). All NMR data were acquired in 20 mM Na_2_HPO_4_ (pH = 7.4) at 25 °C and ^15^N-Spike-RBD amide chemical shift assignments were from BMRB entry 50809^40^. The amides highlighted in *B* are bounded by a box in *A* with the residues labeled. In *C*, ± is the fitted standard error. Titrant concentrations (in µM) were 0 (blue), 50 (cyan), 100 (green), 150 (black), 300 (orange) and 500 (red).

Since our MST and steady-state fluorescence experiments relied on fluorophore-tagged proteins and the SEC-MALS did not demonstrate co-elution using unmodified proteins, we next turned to solution NMR spectroscopy to confirm the interactions using untagged proteins and to map the interaction sites at the residue level. First, the ^1^H-^15^N HSQC spectrum of ^15^N-Spike-RBD was acquired in the absence and presence of increasing concentrations of unlabeled Anx5 target to estimate affinity and probe the interaction site on Spike-RBD. The ^1^H-^15^N HSQC NMR spectrum obtained with ~ 150 µM ^15^N-Spike-RBD alone showed highly dispersed cross-peaks (^1^H: ~6.0–10.0 ppm) consistent with a folded protein conformation and previous ^1^H-^15^N HSQC data for this domain (Fig. 5A)^40^. The addition of increasing Anx5 concentrations to ^15^N-Spike-RBD caused broadening of numerous amide (^1^H-^15^N) crosspeaks and chemical shift positional changes for a few number of peaks, confirming the direct interactions between these unmodified proteins. A simultaneous one-site binding fit of the total ^1^H and ^15^N chemical shift perturbation (CSP) of 4 peaks showing positional changes revealed a K_D_ of 0.38 ± 2 µM (Fig. 5B and C).

Since the Spike-RBD ^1^H-^15^N chemical shift assignments under our solution conditions are known (BMRB entry 50809) and the most prominent spectral change was peak broadening, we plotted the relative loss in peak intensity at each residue position (Fig. 5D). Mapping the intensity (I) ratio [i.e. ^15^N-Spike-RBD: Anx5 (I)/^15^N-Spike-RBD (I_0_)] on the Spike-RBD structure revealed global resonance broadening, as expected given the formation of an ~ 63 kDa complex. Nevertheless, the most enriched region of intensity loss was on the Spike-RBD interface that engages ACE2, whereas the distal face remained the least affected (Fig. 5E, Fig. S3A and B). In other words, Anx5 primarily interacts with Spike-RBD at the same interface that engages ACE2.

**Fig. 6:**
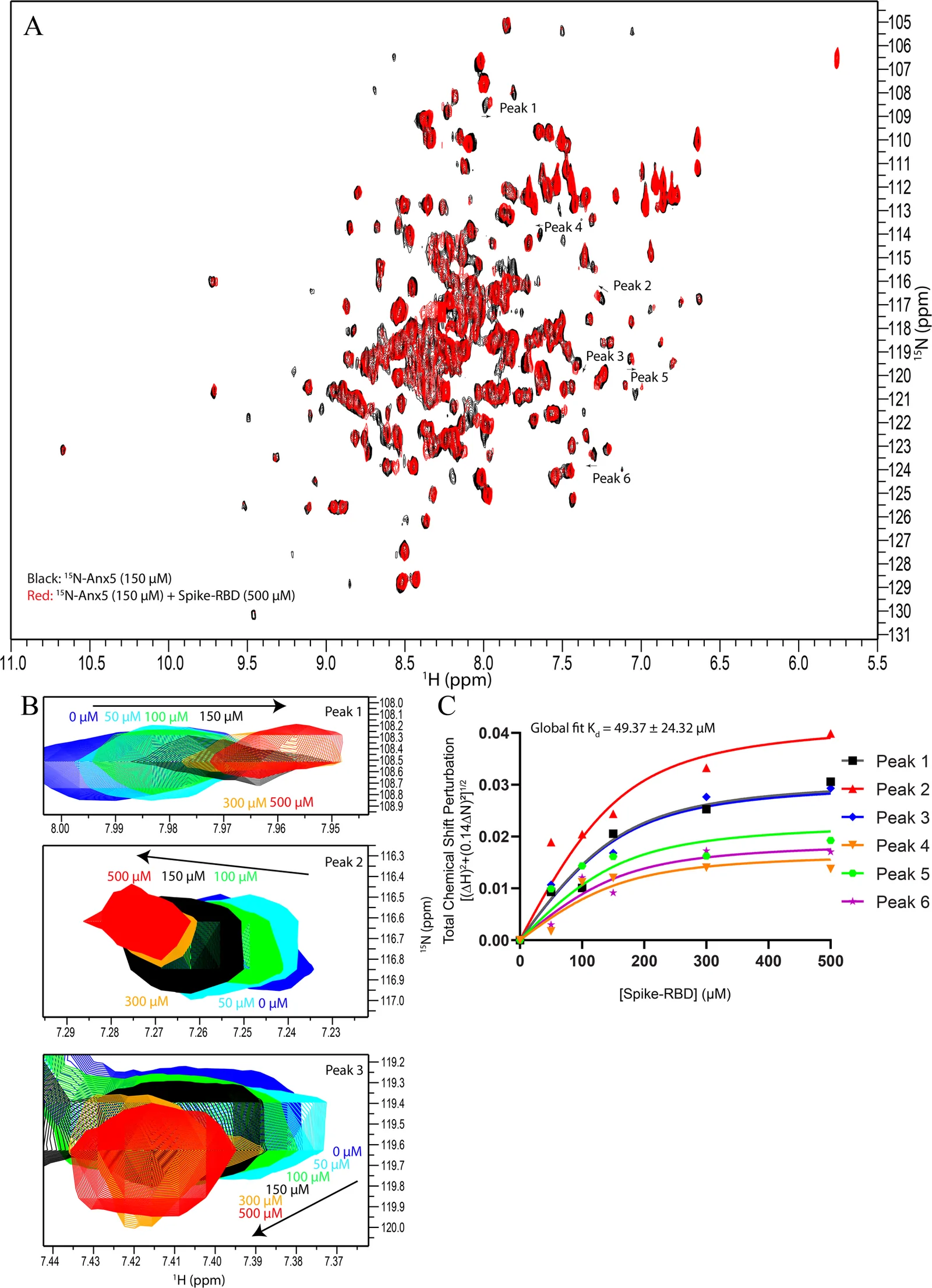
Solution NMR analysis of ^15^N-Anx5 interactions with Spike-RBD. (**A**) Overlaid ^1^H-^15^N HSQC NMR spectra of free (apo) ^15^N-Anx5 (~ 150 µM, black) and ^15^N-Anx5 (~ 150 µM) in complex with Spike-RBD (~ 500 µM, red). (**B**) Zoomed views of representative ^15^N-Anx5 amide crosspeaks undergoing fast exchange CSPs as a function of increasing Spike-RBD concentration. (**C**) Simultaneous fits of ^15^N-Anx5 amide crosspeak CSPs to a one-site binding model taking into account protein concentration, extracting a global K_D_ = 49.4 ± 24 µM. All NMR data were acquired in 20 mM Na_2_HPO_4_ (pH = 7.4) at 25 °C. The amide CSPs highlighted in *B* are indicated with arrows and labeled in *A*. In *C*, ± is the fitted standard error. Titrant concentrations (in µM) were 0 (blue), 50 (cyan), 100 (green), 150 (black), 300 (orange) and 500 (red).

We also prepared uniformly labeled ^15^N-Anx5 and acquired an ^1^H-^15^N HSQC spectrum. Despite the large size of Anx5 for conventional solution NMR (i.e. >30 kDa), the spectrum showed well-dispersed ^1^H-^15^N amide crosspeaks over the ~ 6–10 ppm range, consistent with a folded conformation (Fig. 6A). Given the high quality ^15^N-Anx5 ^1^H-^15^N HSQC spectrum, we next titrated unlabeled Spike-RBD into ^15^N-Anx5 and monitored interactions through changes in ^1^H-^15^N HSQC spectra. Consistent with our ^15^N-Spike-RBD titration with Anx5, this reverse titration showed broadening of numerous ^15^N-Anx5 amide crosspeaks with increasing Spike-RBD concentrations as well as some small positional changes for a few crosspeaks (Fig. 6B). A simultaneous one-site binding fit of the total ^1^H and ^15^N CSPs of 6 peaks showing positional changes revealed a K_D_ of 49.4 ± 24 µM (Fig. 6C); however, simultaneous fitting of the apparently 3 tightest binding residues showed a K_D_ of 6.5 ± 8.5 µM (Table S1).

Thus, the Anx5 interaction with Spike-RBD occurs with ~ µM affinity, engaging the viral protein at the same interface as ACE2. Note that ^15^N-Anx5 chemical shift assignments are not available, precluding the mapping of the interface on Anx5.

### Anx5 interacts with ACE2 with µM affinity

**Fig. 7:**
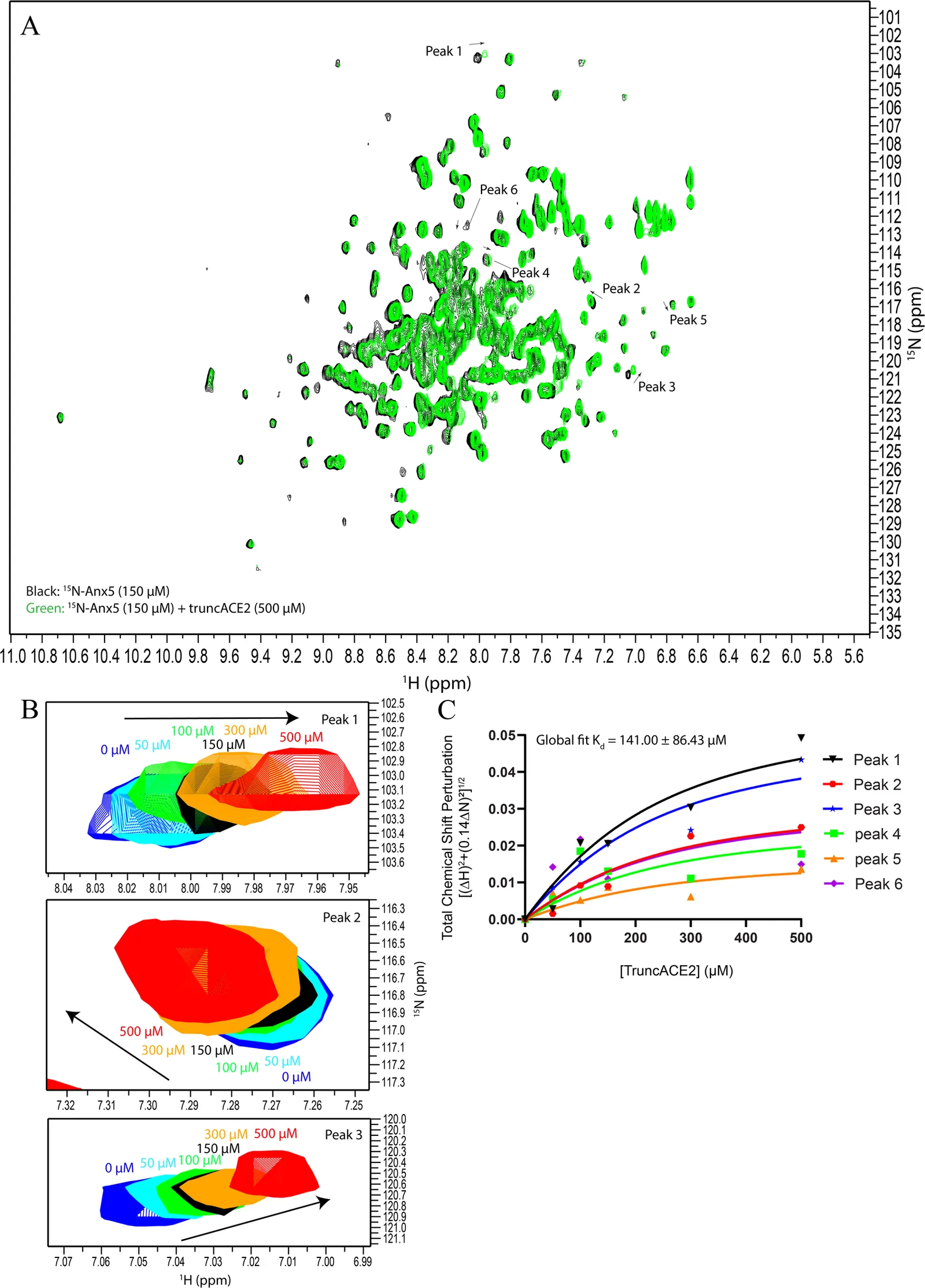
Solution NMR analysis of ^15^N-Anx5 interactions with truncACE2. (**A**) Overlaid ^1^H-^15^N HSQC NMR spectra of free (apo) ^15^N-Spike-RBD (~ 150 µM, black) and ^15^N-Anx5 (~ 150 µM) in complex with truncACE2 (~ 500 µM, green). (**B**) Zoomed views of representative ^15^N-Anx5 amide crosspeaks undergoing fast exchange CSPs as a function of increasing truncACE2 concentration. (**C**) Simultaneous fits of ^15^N-Anx5 amide crosspeak CSPs to a one-site binding model taking into account protein concentration, extracting a global K_D_ = 141 ± 87 µM. All NMR data were acquired in 20 mM Na_2_HPO_4_ (pH = 7.4) at 25 °C. The amide CSPs highlighted in *B* are shown with arrows in *A*. In *C*, ± is the fitted standard error. Titrant concentrations (in µM) were 0 (blue), 50 (cyan), 100 (green), 150 (black), 300 (orange) and 500 (red).

Since the maximum attainable ACE2 concentration was ~ 0.7-1 mg/mL but higher for truncACE2, and both ACE2 and truncACE2 are refractory to detection using conventional solution NMR experiments due to MWs of ~ 71 and 46 kDa, respectively, we probed interactions of unlabeled truncACE2 with ^15^N-Anx5 in ^1^H-^15^N HSQC experiments. As expected, increasing concentrations of truncACE2 lead to more than a dozen ^1^H-^15^N crosspeaks showing severe broadening in the ^15^N-Anx5 ^1^H-^15^N HSQC spectra, indicating interactions between the untagged proteins (Fig. 7A). A few amides showed small positional changes, which were used to construct binding isotherms and extract an affinity for the interaction (Fig. 7B and C). Simultaneous one-site binding fit of the total ^1^H and ^15^N CSPs of 6 peaks showing positional changes revealed a K_D_ of 141 ± 86 µM (Fig. 7C); however, simultaneous fitting of the apparently 3 tightest binding residues showed a K_D_ of 3.4 ± 13 µM (Table S1). Therefore, Anx5 also interacts with ACE2 with ~ µM affinity, and taken together with our Spike-RBD experiments, these results highlight the dual-targeting of Anx5 across the SARS-CoV-2:host-cell attachment interface.

### Anx5 competes with ACE2 for the same binding interface on Spike-RBD

**Fig. 8:**
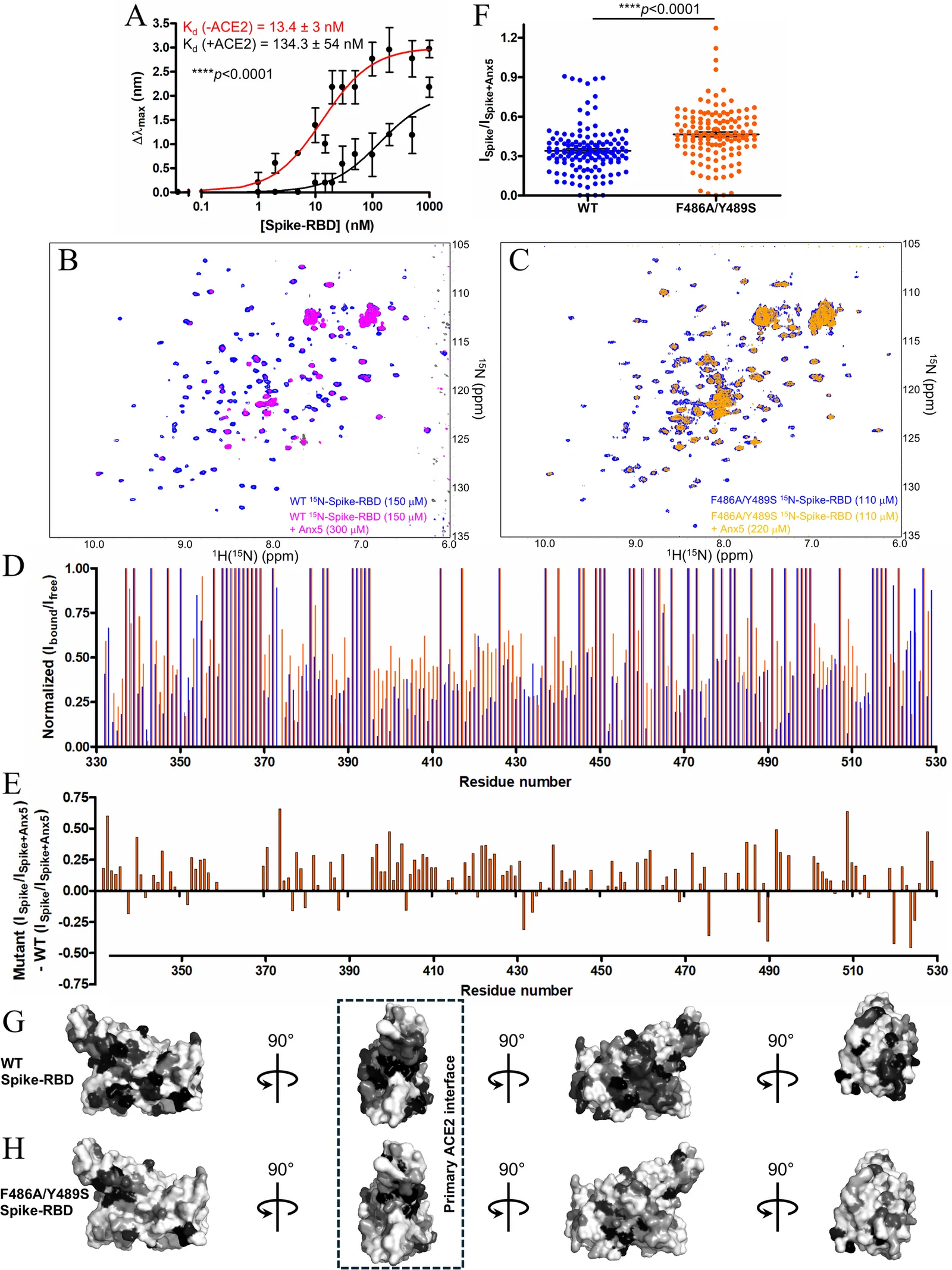
Specificity of Anx5 interactions with Spike-RBD. (**A**) Competitive binding assay of Anx5 (10 nM) and wild-type (WT) Spike-RBD in the absence (red) or presence (black) of 100 nM ACE2. Data were fitted to a one-site binding model, and an *F*-test confirms a significant ~ 10-fold weakening of the apparent affinity in the presence of ACE2, with K_D_ values of 134 nM versus 13.4 nM, respectively (****p < 0.0001). (**B**) Overlaid ^1^H-^15^N HSQC NMR spectra of free 150 µM WT ^15^N-Spike-RBD (blue) and the complex after 300 µM Anx5 addition (1:2 molar ratio; magenta). Extensive peak broadening and signal attenuation indicate a robust interaction. (**C**) Overlaid ^1^H-^15^N HSQC NMR spectra of free 102 µM F486A/Y489S ^15^N-Spike-RBD (blue) and the complex after 202 µM Anx5 addition (1:2 molar ratio; orange). The double mutation shows less signal intensity loss compared to the WT complex. (**D**) Residue-specific intensity (I) ratios (I_bound_/I_free_) for WT (blue) and the F486A/Y489S mutant (orange) ^15^N-Spike-RBD in the presence of Anx5. All amide cross-peak intensities were normalized to the C-terminal amide within the respective apo-state spectra (I_free_). (**E**) Residual intensity plot depicting the difference in attenuation profiles (I_bound_/I_free_, mutant – I_bound_/I_free_, WT). Overwhelmingly positive values across the sequence provide evidence of reduced signal attenuation in the double mutant. (**F**) Comparison of all observable amide cross-peak intensity ratios. The F486A/Y489S population exhibits a significantly higher mean intensity retention versus WT (****p < 0.0001). (**G**) Anx5-induced residue-specific I_bound_/I_free_ values for WT mapped onto the Spike-RBD structure (PDB: 6LZG.pdb). The grey-scale gradient corresponds to ratios from 0.75 (white) to 0.25 (black). (**H**) Anx5-induced residue-specific I_bound_/I_free_ values for the F486A/Y489S mutant mapped onto the Spike-RBD structure. The dashed box denotes the primary ACE2 interface, highlighting the loss of localized broadening relative to the WT interface.

To assess whether Anx5 exerts inhibitory effects by occluding the ACE2 interface of Spike-RBD, we next performed a competitive binding assay monitored by steady-state fluorescence. Spike-RBD was titrated against fluorescein-labeled Anx5 (10 nM) in the presence and absence of a 10-fold molar excess of ACE2 (100 nM). To circumvent any dilution-dependent normalization, we monitored shifts in the fluorescence emission maximum rather than absolute intensity. In the presence of ACE2, the apparent affinity of Anx5 for Spike-RBD was reduced ~ 10-fold (i.e. K_D_ increased from 13.4 nM to 134 nM; *p* < 0.0001), suggesting that Anx5 engages an interface that overlaps with or is sterically proximal to the ACE2-binding site (Fig. 8A).

To further characterize the interactions, we generated a double-mutant Spike-RBD (F486A/Y489S) protein construct. We created the F486A/Y489S variant based on past work showing these mutations result in ACE2 binding-deficiency concomitant with increased Spike-RBD expression^41^. We reasoned that if Anx5 binding is specific to the Spike-RBD: ACE2 interface, these mutations that disrupt ACE2 interactions should also significantly attenuate the interaction with Anx5. ^1^H-^15^N HSQC NMR spectra of the wild-type (WT) ^15^N-Spike-RBD: Anx5 complex exhibited extensive peak broadening and signal attenuation, characteristic of a robust macromolecular interaction (Fig. 8B). In contrast, the ^15^N-Spike-RBD F486A/Y489S mutant showed significantly less peak broadening at the same 1:2 molar ratio (Fig. 8C). Quantitative analysis of residue-specific intensity ratios (I_bound_/I_free_) confirmed that the mutant retained significantly higher signal intensity across the sequence compared to WT (Fig. 8D and F; *p* < 0.0001). Notably, when these ratios were mapped onto the Spike-RBD structure, the localized broadening observed at the ACE2-binding interface in the WT protein was largely diminished in the mutant (Fig. 8G and H). Collectively, these competition and mutagenesis data demonstrate that Anx5 directly competes with ACE2 by targeting a shared binding footprint on the Spike-RBD.

### Anx5 inhibits SARS-CoV-2 entry in a time-dependent manner and improves host cell survival

**Fig. 9:**
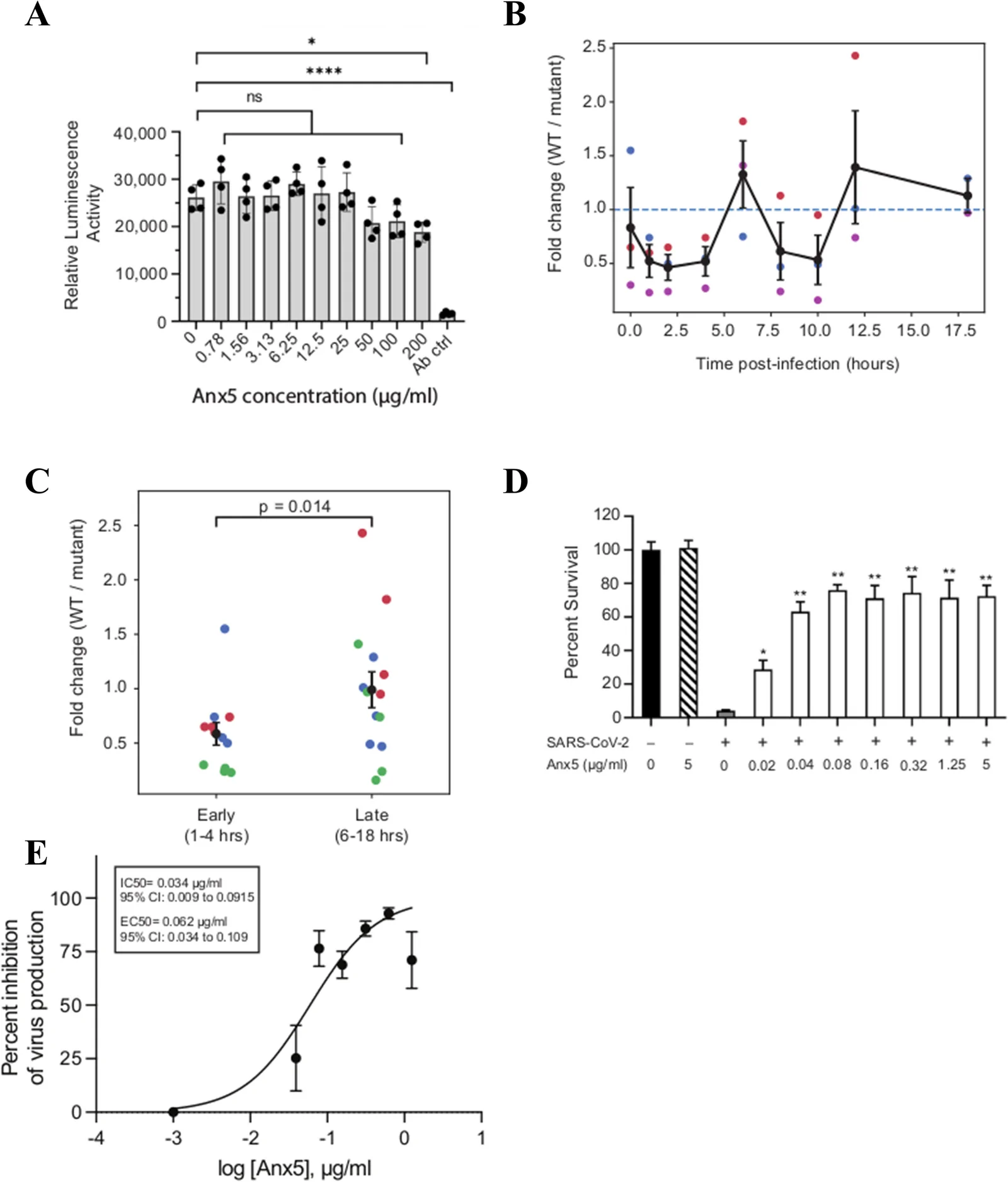
Effects of recombinant human Anx5 on potential SARS-CoV-2 pathogenicity. (**A**) SARS-CoV-2 pseudoviral entry in 293T-ACE2 cells in the presence of increasing recombinant human Anx5 concentration. Lentiviral pseudoviruses expressing SARS-CoV-2 Spike were incubated with Anx5 for 1 h before infecting cells. Luminescence was evaluated 48 h post-infection. Ab ctrl represents a positive control for entry inhibition using convalescent COVID-19 patient serum. (**B**) Time-of-addition analysis showing extracellular viral RNA levels following treatment with wild-type Anx5 at the indicated timepoints post-infection (0, 1, 2, 4, 6, 8, 10, 12, and 18 h). Data are normalized to a Ca²⁺-binding-deficient Anx5 mutant control and presented as fold change [wild-type (WT)/mutant]. Individual biological replicates are color matched to replicates (*n* = 3, except 18 h where *n* = 2) and shown with mean ± SEM (black colored datapoints). The dashed line indicates the mutant control baseline (fold-change = 1.0). (**C**) Early (1–4 h) versus late (6–18 h) timepoints grouped to assess temporal effects. Each point represents a biological replicate matched by color and shown with mean ± SEM. Statistical comparison was performed using Welch’s two-sided t-test. (**D**) SARS-CoV-2-infected 293T-ACE2 cell survival as a function of increasing recombinant human Anx5 concentration. 293T-ACE2 cells were infected with SARS-CoV-2 at an MOI of 0.01. Percent cell survival was measured at 72 h post-viral infection and Anx5 treatment. (**E**) Dose-dependent inhibition of SARS-CoV-2 production by recombinant human Anx5 measured by RT–qPCR. 293T-ACE2 cells were infected with SARS-CoV-2 and treated with increasing concentrations of Anx5. At 72 h post-infection, viral RNA released into the culture supernatant was quantified by RT–qPCR and expressed as percent inhibition of virus production relative to untreated infected controls. Data were fit using nonlinear regression (log[inhibitor] vs. response, variable slope) to calculate inhibitory parameters. Values represent mean ± SEM from 4 independent experiments. In *A and B*, data are means ± SEM of *n* = 3–4 experiments. Statistics are one-way ANOVA followed by Dunnett’s post-hoc with comparisons to the untreated group and *p ≤ 0.05, ** *p* ≤ 0.01, ****p ≤ 0.0001 considered significant. ns, not significant.

Having found that Anx5 binds to both Spike-RBD and ACE2, we next assessed how this binding affects SARS-CoV-2 entry and survival of cells expressing ACE2. We used HEK-293T cells, stably expressing human ACE2 for these assays (293T-ACE2). To evaluate viral entry, we pre-incubated 293T-ACE2 cells with 0–200 µg/mL Anx5 for 1 h prior to adding lentiviral pseudoviruses expressing SARS-CoV-2 Spike. Viral entry was assessed by measuring luciferase activity expressed from the pseudovirus. Cells treated with Anx5 showed a significant reduction in luminescence, indicating decreased pseudoviral entry (Fig. 9A). However, this inhibitory effect was only evident at the highest Anx5 concentration tested (200 µg/mL, ~ 6 µM). As a positive control, incubation with convalescent COVID-19 patient serum resulted in near-complete inhibition of entry, with luciferase activity reduced to background levels. The ~ µM magnitude of entry inhibition is consistent with the ~ µM binding affinities measured between Anx5 and Spike-RBD/ACE2 by NMR and MST, supporting a coherent mechanistic framework. The 293T-ACE2 system was used as a controlled, receptor-defined model to determine whether dual engagement of Spike and ACE2 could translate into measurable effects on ACE2-dependent entry. Such systems are widely used to study SARS-CoV-2 attachment and entry under defined receptor conditions^7,42^, and here are interpreted as mechanistic proof-of-principle rather than a measure of physiological potency. While physiologically relevant respiratory epithelial models will be required to determine the generalizability of the findings, the present experiments were designed to establish whether direct engagement of the Spike-RBD: ACE2 interface by Anx5 is sufficient to inhibit ACE2-dependent viral entry under controlled receptor-defined conditions.

To define the stage of the viral lifecycle affected by Anx5, we performed time-of-addition experiments in 293T-ACE2 cells infected with SARS-CoV-2. Viral RNA released into the supernatant was quantified by qPCR following treatment with wild-type Anx5 added at defined timepoints post-infection (0–18 h). All measurements were normalized to a functionally inactive Anx5 mutant deficient in Ca^2+^ and phosphatidylserine (PS) binding (E72Q/D144N/E228Q/D303N^43,44^) to control for nonspecific protein effects. Early addition of wild-type Anx5 resulted in a marked reduction in extracellular viral RNA relative to the mutant control (Fig. 9B). The strongest effect was observed when Anx5 was added within the first 2 h post-infection, with an ~ 50–55% decrease in viral RNA levels (mean fold-change ~ 0.46 at 2 h). A similar magnitude of reduction was observed at 1 and 4-hrs post-infection (mean fold-change ~ 0.52), although with greater variability between biological replicates. Some variability was observed at intermediate timepoints (i.e. 6 h), consistent with transitional infection dynamics. In contrast, when Anx5 addition was delayed to 6 h or later, the early inhibitory effect was lost, and viral RNA levels were not consistently reduced relative to the mutant control, with several timepoints approaching or exceeding baseline (i.e. mean fold-change ~ 1.33 at 6 h and ~ 1.39 at 12 h).

Statistical analysis using two-sided one-sample t-tests relative to the mutant-normalized baseline (fold-change = 1.0) identified the strongest evidence of inhibition at 2 h post-infection (*p* = 0.046, unadjusted), with early timepoints (1–4 h) consistently trending toward reduced viral RNA (1 h: *p* = 0.089; 4 h: *p* = 0.072) (Fig. 9B). However, these effects did not remain statistically significant following Holm correction for multiple comparisons (2 h: adjusted *p* = 0.42), reflecting variability across biological replicates and limited sample size. To directly assess temporal dependence, early (1–4 h) and late (6–18 h) timepoints were grouped. Early treatment significantly reduced extracellular viral RNA compared to late treatment (Welch’s two-sided t-test, *p* = 0.014), an effect also supported by a Mann-Whitney U test (*p* = 0.037), indicating a temporally restricted antiviral effect (Fig. 9C).

To assess whether inhibited viral entry correlates with protection from SARS-CoV-2 infection, we also assessed cell survival of 293T-ACE2 cells infected with SARS-CoV-2. In these survival assays, 293T-ACE2 cells were pre-treated with 0–5 µg/mL Anx5 for 2 h, followed by a 1 h incubation with SARS-CoV-2. After infection, cells were washed and cultured for an additional 72 h in the continued presence of Anx5. Notably, all tested Anx5 concentrations (0.02 to 5 µg/mL) resulted in a significant increase in cell survival, as assessed using the CellTiter Glo 2.0 assay (Fig. 9D). These experiments were performed in complete culture medium containing serum-derived proteins, providing a high background of unrelated extracellular proteins. Despite this protein-rich environment, Anx5 produced a clear and dose-dependent functional profile, arguing against nonspecific effects from protein addition alone. To further evaluate whether this survival benefit was associated with altered infection dynamics, we quantified SARS-CoV-2 RNA released into the culture supernatant by RT-qPCR. Anx5 treatment resulted in a clear dose-dependent reduction in extracellular viral RNA, with a half-maximal inhibitory concentration (IC_50_) of 0.034 µg/mL and half-maximal effective concentration (EC_50_) of 0.062 µg/mL (Fig. 9E). Since these measurements quantify viral genomes present in the supernatant rather than intracellular RNA, they reflect the net viral output from infected cultures. This reduction in released viral RNA indicates that Anx5 decreases viral burden at the level of virus production and/or spread. The alignment between decreased viral output and improved cell viability supports the interpretation that enhanced survival is associated with reduced infection pressure rather than cytoprotection alone.

Collectively, these functional assays demonstrate that Anx5 both hinders viral entry and enhances the survival of ACE2-expressing mammalian cells. The time-of-addition analysis indicates that the antiviral effect is restricted to early stages of infection, consistent with a mechanism involving interference with viral entry. Notably, the concentration required to inhibit pseudoviral entry (~ 6 µM) closely matches the µM-range affinities measured by MST and NMR, whereas improvements in cell survival and reductions in extracellular viral RNA were observed at substantially lower concentrations. These findings indicate that the effects observed in the pseudoviral entry and live-virus assays occur over different concentration ranges and likely reflect distinct biological consequences of Anx5 treatment.

## Discussion

**Fig. 10:**
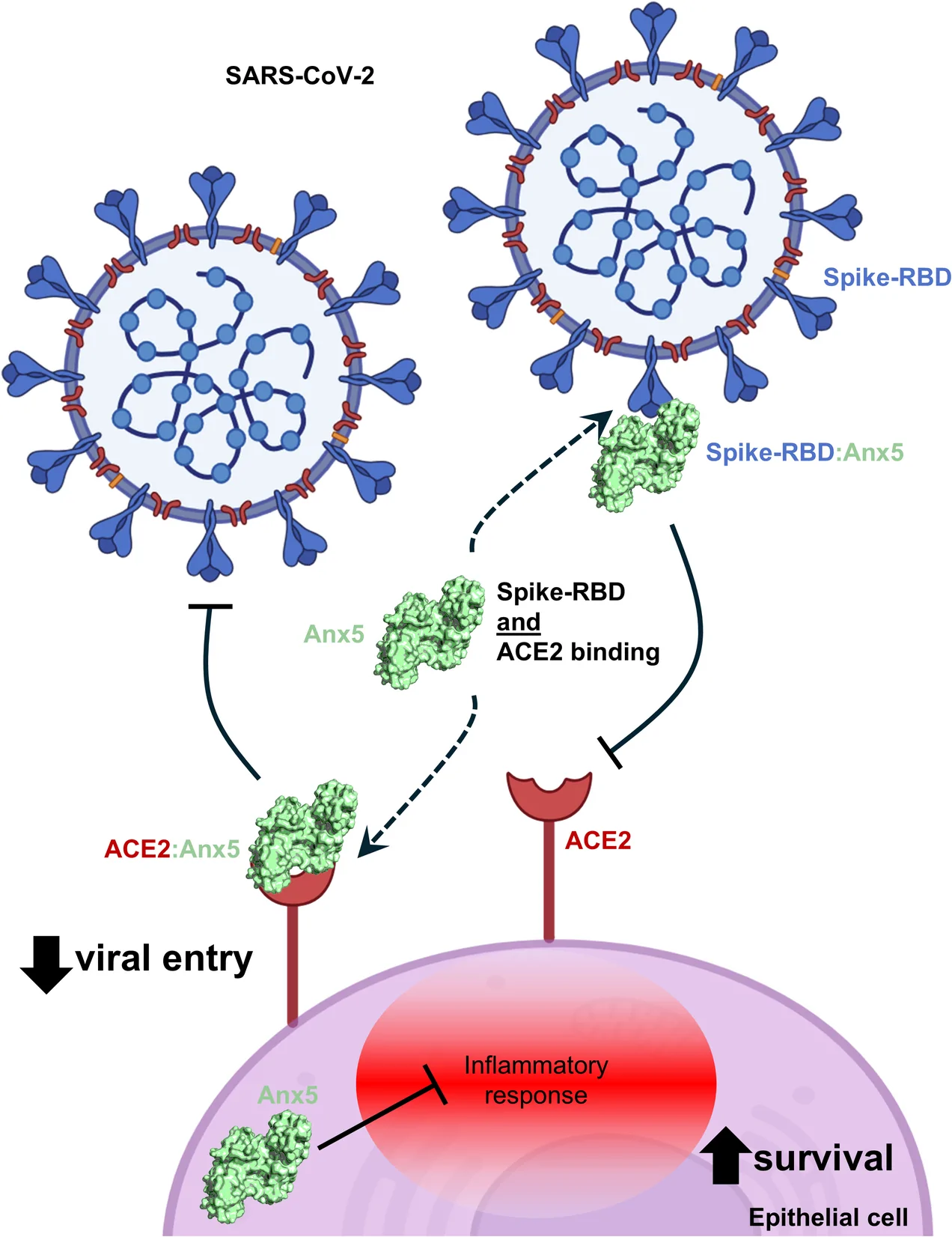
Proposed model of Anx5 activity against SARS-CoV-2. At µM concentrations, Anx5 directly binds both Spike-RBD and ACE2, competitively interfering with the Spike-RBD: ACE2 interaction and reducing viral entry. Independently, lower concentrations of Anx5 improve cell survival and reduce extracellular viral burden through mechanisms that remain incompletely defined and may involve phosphatidylserine-dependent, membrane-associated, or host-directed protective activities (e.g. inflammatory responses^21^). The latter mechanism is proposed based on functional observations but was not directly tested in the present study.

Anx5 reduces pro-inflammatory cytokine expression and improves survival in preclinical models of sepsis^21^. Further, randomized, placebo-controlled Phase II clinical trials have shown feasibility and safety of Anx5 in the treatment of patients with sepsis (NCT 04898322) and severe COVID-19^35,36^. To gain insights into the molecular basis underlying Anx5 protection against COVID-19, we expressed and isolated SARS-CoV-2 Spike-RBD, human ACE2, and human Anx5 proteins (Fig. 1). We found Anx5 binds to both Spike-RBD and ACE2 (Figs. 3 and 4). SEC-MALS indicated a fast-exchange binding equilibrium between Anx5 and both ACE2 and Spike-RBD with no complexed co-elution evident (Fig. S2). Nevertheless, solution NMR confirmed dual binding of Anx5 with both Spike-RBD and ACE2 and notably, showed that the Anx5 binding footprint on Spike-RBD directly overlaps with the same region critical for ACE2 engagement (Figs. 5, 6 and 7 and Fig. S3). This interface was validated by site-directed mutagenesis of the ACE2-binding footprint on Spike-RBD, which significantly attenuated Anx5-induced peak broadening and was consistent with an observed direct competition between Anx5 and ACE2 for Spike-RBD binding (Fig. 8). Functionally, Anx5 was found to inhibit SARS-CoV-2 pseudoviral entry into ACE2 overexpressing HEK-293T cells at ~ µM concentration, but improved survival of these cells infected with SARS-CoV-2 at ~ nM levels (Fig. 9). Together, these findings suggest that Anx5 exerts both direct effects on viral entry and additional biological effects that contribute to reduced viral burden and improved cell survival during infection (Fig. 10).

In a number of studies, Spike-RBD has been shown to interact tightly with ACE2, exhibiting K_D_s in the ~ 5-150 nM range using different approaches^9,10,12–16^. ACE2 metallopeptidase is a host cell entry receptor for several coronaviruses (CoVs), including SARS-CoV, HCoV-NL63, and SARS-CoV-2^45^. The loss of ACE2 activity post-SARS-CoV-2 entry and shedding of the receptor prevents the production of angiotensin (Ang) 1–7 and 1–9, and leads to upregulation of Ang II, resulting in vasoconstriction, microthrombosis, thrombophilia, alveolar epithelial injury, and respiratory failure^46^. Indeed, Ang II and Ang II type I receptor (AT1R) signaling is known to induce macrophage activation syndrome (MAS) and ARDS through activation of macrophages and release of inflammatory cytokines^46,47^. Further, in severe COVID-19 patients, increased levels of circulating ACE2 was found to be associated with serious cardiovascular complications such as aortic stenosis and heart attack^48^. Thus, inhibition of CoV interaction with ACE2 is key to preventing the loss of ACE2 protective functions at epithelial and endothelial sites. Here, we observed binding of Anx5 to ACE2 and Spike-RBD, and remarkably, our solution NMR experiments indicate that Anx5 binds to Spike-RBD at the same site that engages ACE2, implying that Anx5 acts as a dual-action inhibitor by sterically occluding the binding surfaces of both the virus and host receptor.

Interestingly, Anx5 treatment inhibited SARS-CoV-2 pseudoviral entry into HEK-293T cells overexpressing ACE2 (293T-ACE2). Notably, Anx5 also enhanced the survival of 293T-ACE2 cells following infection with live SARS-CoV-2 at much lower concentrations (~ nM) than those required to block the pseudoviral entry (~µM). These findings suggest that Anx5 may exert protective effects through more than one mechanism. First, based on our binding, viral entry and time-of-addition data, Anx5 likely interferes with viral attachment by binding Spike-RBD and/or ACE2, thereby preventing ACE2-dependent entry. Second, the enhanced survival observed at lower concentrations raises the possibility of an additional protective effect that is independent of viral entry inhibition (Fig. 10). Thus, while µM concentrations are required to directly interfere with Spike-RBD: ACE2 engagement and inhibit viral entry, the lower-concentration effects observed in the live-virus experiments are unlikely to be explained solely by direct occupancy of the Spike-RBD or ACE2 binding interfaces. Since extracellular viral RNA was measured after prolonged infection, this endpoint reflects the cumulative outcome of viral replication, spread, and cell survival rather than viral entry alone. Consequently, modest effects occurring during the earliest stages of infection may become amplified over multiple rounds of viral replication and cell-to-cell spread.

Although we did not directly assess immune activation, our observations are consistent with prior findings showing that Anx5 can reduce inflammatory injury. Specifically, Anx5 suppresses pro-inflammatory cytokine expression by sequestering externalized phosphatidylserine^23^. Further, Anx5 inhibits TLR4 signaling, which is highly relevant given that Spike-RBD directly triggers TLR4-mediated inflammatory cascades^22,49^. In further support of this premise, extracellular vesicles derived from the plasma of COVID-19 patients, which are known to induce endothelial cell death and upregulate adhesion molecules such as P-selectin and VCAM-1, were rendered non-cytotoxic by pre-treatment with low concentrations of Anx5 (0.07 nM)^50^. These vesicles are known to contribute to neutrophil adhesion and neutrophil extracellular trap (NET) formation, key features of COVID-19-associated vascular inflammation. Notably, the dose-dependent survival benefits we observed in serum-rich media further support the specificity of this effect over non-specific protein interference. Collectively, these observations are consistent with the possibility that Anx5 exerts additional protective effects beyond direct inhibition of viral entry.

The time-of-addition analysis provides important insight into the stage-specific activity of Anx5 during SARS-CoV-2 infection. The observation that inhibition is restricted to early timepoints, with no effect when treatment is delayed, indicates that Anx5 acts during the initial stages of infection, likely at or prior to viral entry. This temporal restriction is consistent with the ability of Anx5 to interact with Spike and ACE2 and suggests that antiviral activity is not mediated through effects on viral replication, assembly or release. The requirement for an intact Ca^2+^-dependent phospholipid-binding function further supports a membrane-associated mechanism, potentially involving interference with virus-cell attachment or membrane fusion. Further, by binding phosphatidylserine exposed on the viral envelope, Anx5 may block the apoptotic mimicry that SARS-CoV-2 exploits to hijack host entry pathways^51^. Although individual timepoints did not retain statistical significance after correction for multiple comparisons, the consistent reduction observed across early timepoints, together with the significant difference between early and late treatment groups, supports a model in which Anx5 exerts a temporally restricted, function-dependent effect on viral infection. The transient increase observed at ~ 6 h likely reflects variability in viral replication or release dynamics during the transition from entry to productive infection, a stage at which Anx5 no longer exerts consistent inhibitory effects.

It is important to note that our pseudovirus entry assay employed lentiviral particles pseudotyped with the full-length SARS-CoV-2 Spike protein, including both the S1 and S2 subunits. While S1 contains the RBD responsible for ACE2 engagement, S2 mediates viral-host membrane fusion following proteolytic cleavage, typically by TMPRSS2 ^7–10^. As such, the pseudovirus assay reflects multiple stages of viral entry, including receptor binding, membrane fusion, and reporter gene expression, providing a more comprehensive view of entry inhibition than the binding assays using Spike-RBD alone.

Interestingly, other Annexin family members have been implicated in viral infections and inflammation. For example, Annexin A2 (Anx2), a Ca^2+^- and phospholipid-binding protein with structural similarities to Anx5, has been associated with both pro-viral and anti-inflammatory functions^52,53^. In the context of SARS-CoV-2, Anx2 was reported to interact with the Spike glycoprotein and RNA-dependent RNA polymerase, potentially promoting viral attachment, entry, and replication^54^. This Anx2 data contrasts with our findings for Anx5, which appears to interfere with viral entry and enhance cell survival. Yet, Anx2 has also been shown to exert protective effects in sepsis models, where its loss exacerbates disease severity in both mice and humans^55,56^. These observations underscore the complex, context-dependent roles of annexins in host-pathogen interactions and support further exploration of Anx5 as a therapeutic candidate in viral and inflammatory diseases.

One limitation of our study is the large variability in K_D_ values we observed, dependent on the technique used. Specifically, steady-state fluorescence indicated binding affinities of ~ 23–54 nM between Anx5 and targets (ACE2, truncACE2, or Spike-RBD), while MST and NMR showed affinities that were ~ 1–2 order of magnitudes weaker (Table S1). The presence of the Tween 20 detergent in the MST experiments likely contributed to the weaker affinities compared to steady-state fluorescence. Further, the fluorescent tags (RED-NHS and fluorescein) used for the steady-state and MST experiments may have artifactually increased binding affinity compared to NMR by altering hydrophobicity or protein folding. Indeed, the NMR data, which used untagged proteins, showed the interactions were weaker and in fast exchange, also consistent with the SEC data. We note that the high µM concentrations required for NMR may drive secondary associations or lower-affinity binding events, such as those suggested by CSP directional changes at high molar ratios, further contributing to the observed variability in K_D_ values. Overall, we speculate that, while the binding measurements using tagged proteins provide important proof-of-principle data for interactions, the true binding affinity is likely in ~ µM range as reported by NMR. While thermodynamic binding affinity and functional inhibition are distinct parameters, this µM magnitude aligns with the ~ µM concentrations of Anx5 required to inhibit pseudoviral entry in our cellular assays.

Another limitation of this study is that all functional experiments were performed in ACE2-overexpressing HEK-293T cells. While this receptor-defined system is widely used for mechanistic studies of SARS-CoV-2 entry, it does not fully recapitulate the biology of airway epithelial cells that naturally support infection. Consequently, the present work should be interpreted primarily as mechanistic evidence that Anx5 can engage both Spike-RBD and ACE2 and inhibit ACE2-dependent entry. Future studies using physiologically relevant respiratory epithelial models, primary airway epithelial cultures, or organoid systems, will be important to establish the broader physiological relevance of these findings.

In conclusion, we demonstrate that Anx5 binds to ACE2 and Spike-RBD, reduces viral entry and enhances survival of SARS-CoV-2 infected mammalian cells, highlighting potential multi-pronged therapeutic benefit by Anx5 in SARS-CoV-2 pathogenesis (Fig. 10). Given the interaction with the ACE2 receptor and distinct intracellular pro-survival effects, Anx5 could theoretically remain effective against both current and emerging SARS-CoV-2 variants. Importantly, a key advantage of Anx5 is its established safety profile for therapeutic use in humans^35,57^.

## Methods

### General information

All chemicals and salts were purchased from BioShop or Fisher Chemical. Optical density at 600 nm (OD_600_) was measured on a spectrophotometer (Eppendorf). Protein concentrations were determined on a DeNovix DS-11 spectrophotometer using UV-Vis absorbance measurements at 280 nm. Protein purity and identity were confirmed by SDS-PAGE analysis (15% (w/v) SDS-PAGE gel with Coomassie blue staining) and electrospray ionization mass spectrometry (ESI-MS) on QTof Ultima mass spectrometer (Waters) equipped with a Z-spray source and run in positive ion mode with an Agilent 1100 HPLC used for flow injection. Replicates within each experiment were performed using different batch recombinant proteins.

### Protein expression

The cDNA region encoding the SARS-CoV-2 Spike-RBD (residues 319–541, NCBI accession # 7FG7_A), human ACE2 extracellular domain (residues 19–615, NCBI accession # BAB40370), and truncated human ACE2 (truncACE2, residues 19–398, NCBI accession # BAB40370) were cloned separately into pET-28a vector (Novagen) at NheI and XhoI restriction sites. Human annexin A5 (residues 1-320, NCBI accession # NP_001145) cloned into pEX-N-His (Blue Heron Biotech). The plasmids were transformed separately into BL21-CodonPlus (DE3) *Escherichia coli* (*E. coli*) competent cells and cultured in 20 ml Luria broth (LB) medium containing kanamycin (60 µg/ml, pET-28a plasmids) or ampicillin (100 µg/ml, pEX-N-His plasmid) on a shaker (200 rpm) at 37 °C for ~ 16 h. The cells were then cultured in 1 L LB up to an OD_600_ of 0.6–0.8, and expression was induced with isopropyl β-d-1-thiogalactopyranoside (IPTG, Wisent Bio Products; Spike-RBD and ACE2: 300 µM; Anx5: 500 µM; truncated ACE2: 1000 µM) and cultured overnight on a shaker (200 rpm) at 23.5 °C (Spike-RBD), 25 °C (ACE2 and truncated ACE2), or 18 °C (Anx5). The cells were harvested by centrifuging at 7250 × *g* for 30 min at 8 °C, and the pellets were frozen at -80 °C. ^15^N-labeled-Spike-RBD and ^15^N-labeled-Anx5 were similarly obtained by expressing the cells in 1 L M9 minimal media (pH 7.4) supplemented with 1 mg biotin, 50 µM thiamine, 1 g ^15^N-NH_4_Cl (Sigma) as the sole nitrogen source, and the appropriate antibiotic.

### Protein extraction and purification

*Spike-RBD and* ^15^*N-Spike-RBD*: The cell pellet was suspended in 45 ml lysis buffer [6 M guanidine hydrochloride (Gdn.HCl), 20 mM Tris.HCl (pH 7.4), 5 mM β-mercaptoethanol (β-ME)] and the homogenized cell suspension was incubated for 90 min on a rotor at room temperature (RT, 20–22 °C). The cell suspension was centrifuged at 10,000 rpm for 40 min at 4 °C and the supernatant was incubated with 1 ml nickel-nitrilotriacetic acid (Ni-NTA HisPur, ThermoFisher Scientific) agarose beads slurry for 90 min on a rotor at RT. His-tagged Spike-RBD protein was extracted off the beads through affinity chromatography as per the Ni-NTA manufacturer’s instructions using the following buffers; wash buffer (3 × 10 ml): 6 M Gdn.HCl, 20 mM Tris.HCl (pH 7.4), 5 mM β-ME, 20 mM imidazole; and elution buffer (8 × 3 ml): 6 M Gdn.HCl, 20 mM Tris.HCl (pH 7.4), 5 mM β-ME, 500 mM imidazole. The protein fractions were refolded in 1 L refolding buffer^39^ (20 mM Na_2_HPO_4_ (pH 7.4), 0.18 mM EDTA, 0.5 M l-arginine, 1.8 mM GSH, 0.9 mM GSSH, 2 M urea) by dropwise dilution and constant stirring at 4 °C overnight. The refolded protein was concentrated to ~ 15 ml using a 10,000 MWCO membrane and buffer was exchanged through dialysis in 1 L 20 mM Na_2_HPO_4_ (pH 7.4) at 4 °C using a 6–8,000 MWCO membrane tubing.

*ACE2 and Truncated ACE2*: The cell pellet was suspended in 45 ml lysis buffer (6 M Gdn.HCl, 20 mM Tris.HCl (pH 7.4), 5% (v/v) glycerol, 0.25 mM EDTA, 5 mM β-ME), and the homogenized cell suspension was incubated for 90 min on a rotor at RT. The cell suspension was centrifuged at 10,000 rpm for 40 min at 4 °C and the supernatant was incubated with 1 ml Ni-NTA agarose beads for 90 min on a rotor at RT. His-tagged ACE2 protein was extracted off the beads through Ni-NTA affinity chromatography; wash buffer (5 × 10 ml): 6 M Urea, 20 mM Tris.HCl (pH 7.4), 5 mM β-ME, 20–50 mM imidazole; and elution buffer (7 × 3 ml): 6 M urea, 20 mM Tris.HCl (pH 7.4), 5 mM β-ME, 300–500 mM imidazole. The protein fractions were dialysed in 1 L refolding buffer (20 mM Tris.HCl (pH 7.4), 150 mM NaCl, 2 mM dithiothreitol (DTT), 5% (v/v) glycerol, 0.1 mM phenylmethylsulfonyl fluoride (PMSF)) at 4 °C using a 50,000 Da (full-length ACE2) or 6–8,000 Da (truncACE2) MWCO membrane tubing. The refolded protein was further purified through an anion-exchange chromatography on an ÄKTA Pure system (Cytiva) using either a HiTrap ANX FF or HiTrap Q FF ion exchange chromatography columns (Cytiva) with 20 mM Tris.HCl (pH 7.4), 2 mM DTT, and 0–2 M NaCl gradient elution over 70 column volumes. The ACE2 fractions were dialysed in 1 L final buffer (20 mM Na_2_HPO_4_ (pH 7.4), 0.1 mM PMSF) at 4 °C using a 50,000 Da (ACE2) or 6–8,000 Da (truncACE2) MWCO tubing.

*Annexin A5 and* ^15^*N-Annexin A5*: The cell pellet was suspended in 45 ml lysis buffer (50 mM Tris.HCl (pH 7.4), 500 mM NaCl, 5% (v/v) glycerol, 1 mM PMSF, and homogenized via sonication on ice. 5 mM β-ME was added to the cell suspension and incubated for 90 min on a rotor at 4 °C. The cell suspension was centrifuged at 10,000 rpm for 40 min at 4 °C and the supernatant was incubated with 1–2 ml Ni-NTA agarose beads for 90 min on a rotor at RT. His-tagged Anx5 protein was extracted off the beads through Ni-NTA affinity chromatography; wash buffer (3 × 10 ml): 50 mM Tris.HCl (pH 7.4), 500 mM NaCl, 5% (v/v) glycerol, 5 mM β-ME, 20 mM imidazole; and elution buffer (8 × 2 ml): 50 mM Tris.HCl (pH 7.4), 500 mM NaCl, 5% (v/v) glycerol, 5 mM β-ME, 300 mM imidazole. The protein His-tag was removed through overnight dialysis in 1 L 50 mM Tris.HCl (pH 7.4), 200 mM NaCl, 5% (v/v) glycerol, 1 mM β-ME with His-tagged tobacco etch virus (TEV) protease (0.1 mg TEV per 10 mg protein) at 4 °C using a 6–8,000 MWCO membrane. The TEV protease was removed through a Ni-NTA affinity chromatography (30 min incubation at 4 °C with beads), and the flow through was dialysed in 1 L final buffer (20 mM Na_2_HPO_4_ (pH 7.4)) at 4 °C using a 6–8,000 MWCO membrane tubing.

### Circular dichroism (CD) spectroscopy

Far-UV CD spectra were obtained on a Jasco J-810 Spectropolarimeter connected to a Peltier electronic temperature controller. CD spectra and thermal melts were measured using a 1 mm pathlength quartz cuvette. Each CD spectrum of Spike-RBD (0.4 mg/ml) in 20 mM Na_2_HPO_4_ (pH 7.4) was recorded from 240 to 200 nm at 20 °C with 1 nm data pitch, continuous scanning mode, 20 nm/minute scanning speed, 8 s response, 1 nm bandwidth, and 3 accumulations. Each CD spectrum was corrected with a buffer control spectrum. Thermal melts were acquired at 222 nm CD spectral scans from 20 to 95 °C with 1 °C data pitch, 1 °C/minute temperature slope, 8 s response, and 1 nm bandwidth. The thermal melt data were fitted using a Boltzmann sigmoidal equation with a sloping baseline.

### Fluorescein labeling

Anx5 (200 µg) was precipitated through chloroform-methanol extraction and resuspended in 100 µl of 20 mM Na_2_HPO_4_ (pH 7.4). Sulfhydryl-reactive fluorescein-5-maleimide dye (1 mM, λ_Ex/Em_ 494/519 nm, ThermoScientific) was added to Anx5 solution and incubated at RT for 1 h in the dark. The excess dye was removed through button dialysis in 20 mM Na_2_HPO_4_ (pH 7.4) with three buffer exchanges (3 × 1 L) every 8–24 h. The concentration of fluorescein labeled Anx5 was measured at 497 nm using the fluorescein extinction coefficient (80,000 M^− 1^ cm^− 1^).

### RED-NHS labeling

ACE2 (10 µM) protein buffer was exchanged, and the protein was labeled with primary amine-reactive RED-NHS (λ_Ex/Em_ 650/670 nm) using the Monolith NT Protein Labeling Kit RED-NHS 2nd Generation (NanoTemper) as per the manufacturer’s instructions. The concentration of the labeled protein was measured by absorbance at 280 nm and 650 nm as per the manufacturer’s instructions.

### Steady-state fluorescence

The steady-state fluorescence measurements were carried out with fluorescein labeled protein on a Cary Eclipse Spectrofluorimeter (Varian). Emission scans (500–600 nm) were acquired with an excitation wavelength of 485 nm, excitation and emission slit widths of 5 and 10 nm, respectively, a scan rate of 120 nm min^− 1^, and a photomultiplier tube voltage of 850 V. Fluorescein labeled Anx5 (Anx5-FM) (10 nM, 600 µl) was titrated with increasing concentrations of Spike-RBD or ACE2 in a 1 cm pathlength quartz cuvette at 22.5 °C using 20 mM Na_2_HPO_4_ (pH 7.4) as the experimental buffer. The fluorescence intensities were corrected for the dilution associated with the volume change upon each addition. Fluorescence (F) intensity at each titration point was normalized to initial fluorescence in the absence of titrant (F_0_), as F/F_0_. The resultant binding isotherms were fit to a one-site binding model taking into account protein concentration in GraphPad Prism to extract the equilibrium dissociation constants (K_D_). Control titrations against buffer indicated Anx5-FM adhered to mixing pipette in the absence of ligand. Thus, the first F/F_0_ value was excluded to eliminate the change in fluorescence intensity at the lowest ligand concentration where maximum non-specific adherence occurred. Additionally, the Anx5-FM concentration was set to 8.8 nM and F/F_0_ at 0 ligand concentration was constrained to ≤ 0.88 based on the mean 12% drop in Anx5-FM fluorescence observed in buffer control experiments after the first addition (*n* = 4). Wavelengths at peak intensity maxima (λ_max_) were determined after applying exponential smoothing to the spectra with a dampening factor of 0.95.

### Microscale thermophoresis (MST)

The MST experiments were performed on Monolith NT.115 (NanoTemper Technologies). Fluorescein or RED-NHS labeled target protein (20 nM) with different concentrations of unlabeled ligand protein serially diluted 2-fold in assay buffer (20 mM Na_2_HPO_4_, 0.05% (v/v) tween-20, pH 7.4) were loaded into standard glass Monolith NT.115 Capillaries. MST measurements with Anx5-fluorescein were acquired with Nano BLUE detector using 50% MST Power and high Excitation Power. Measurements with ACE2-RED-NHS were acquired using Nano RED detector with 80% MST Power and medium Excitation Power. All experiments were performed at 22 °C. Thermophoresis was measured as a change in normalized fluorescence (F_norm_ = F_hot_/F_cold_) and the data were analyzed using MO.Affinity Analysis (version 2.3) software (NanoTemper Technologies; https://nanotempertech.com/) to acquire the binding constants (K_D_).

### Size-exclusion chromatography with multi-angle light scattering (SEC-MALS)

SEC-MALS were performed on an ÄKTA Pure using a Superdex 200 Increase 10/300 GL SEC column (Cytiva) in-line with DAWN HELEOS II MALS detector (Wyatt Technology) and Optilab T-rEX differential refractometer (Wyatt Technology) at ~ 10 °C. 20 mM Na_2_HPO_4_ (pH 7.4) was used as the experiment buffer with 100 µl injections of protein samples at appropriate concentrations. SEC-MALS analysis was performed using ASTRA (version 7.1.4) software (Wyatt Technology; https://www.wyatt.com/products/software/astra.html). Protein molecular weights were estimated through Zimm plot analysis with a standard protein refractive index increment (dn/dc) value of 0.185 ml/g, solvent refractive index 1.331 and viscosity 0.8945 cP.

### NMR spectroscopy

NMR experiments were performed on a Varian 600 MHz spectrometer equipped with either an INOVA (Varian) or Bruker console and room temperature HCN triple resonance probes. NMR samples were prepared in 20 mM Na_2_HPO_4_ (pH 7.4), 2.5% (v/v) D_2_O, and 90 µM 4,4-dimethyl-4-silapentane-1-sulfonic acid (DSS). ^1^H-^15^N Heteronuclear Single Quantum Coherence (HSQC) NMR spectra of ^15^N-Spike-RBD or ^15^N-Anx5 samples were recorded at 25 °C using 64 transients, 64 increments in the N dimension, 8,000 Hz ^1^H and 1,800 Hz ^15^N sweep widths, alone or as a function of increasing titrant (i.e. unlabeled truncACE2, unlabeled Anx5 or unlabeled Spike-RBD, as indicated). The ^1^H-^15^N HSQC NMR spectra were processed using the NMRpipe (version 11.7) software (IBBR/UMD; https://www.ibbr.umd.edu/nmrpipe/)^58^ and intensity analysis was performed using Computer Aided Resonance Assignment (version 1.9.1) software (CARA definition team; http://cara.nmr.ch). Chemical shift perturbations (CSPs) were calculated as (ΔH^2^+(0.14×ΔN)^2^)^1/2^, where ΔH is the ppm change in the ^1^H dimension and ΔN is the ppm change in the ^15^N dimension. To extract K_D_ values, CSPs were fit to a one-site binding model taking into account protein concentration. K_D_ was globally shared during simultaneous fits of multiple peak changes. ^15^N-Spike-RBD cross-peaks were assigned by transference using the chemical shifts from BMRB Entry: 50809 ^40^. Cross-peak intensities were extracted and the ratios were calculated as I/I_0_, where I is the intensity of the peaks in the mixture and I_0_ is the intensity of the free (i.e., apo) state. The change in cross-peak intensity ratios in complex with Anx5 was mapped on the Spike-RBD backbone (6LZG.pdb) as a gradient using PyMOL (version 3.1.0) software (Schrodinger, LLC; https://pymol.org/) for visualization.

### SARS-CoV-2 infection

Human 293T cells overexpressing ACE2 (293T-ACE2; BEI Resources, cat: NR-52511) were seeded in a 96-well plate at a density of 1.5 × 10^4^ cells per well for 24 h. Cells were then infected for 2 h with wild-type SARS-CoV-2 (isolate USA-WA1/2020, NR-52281; deposited by the Centers for Disease Control and Prevention and obtained through BEI Resources, NIAID, NIH) in complete Dulbecco’s Modified Eagle Medium (DMEM) at an multiplicity of infection (MOI) of 0.01. This MOI induces near complete cytopathogenicity after 72 h in culture. The virus was pre-treated with varying concentrations of Anx5 (0–5 µg/ml) for 1 h immediately prior to infection. After 2 h infection, the media containing virus was removed and replaced with complete DMEM (400 µl) with or without varying concentrations of Anx5 (0–5 µg/ml) and incubated for 72 h. Post-infection, the cell viability was determined using the CellTiter-Glo 2.0 Cell Viability Assay kit (Promega) according to the manufacturer instructions.

Culture supernatants were collected and clarified by brief centrifugation to remove cellular debris. Viral RNA was isolated from supernatant samples using the MagMAX™-96 Viral RNA Isolation Kit (Life Technologies) according to the manufacturer’s instructions. Eluted RNA was immediately used for RT-qPCR analysis. Quantitative PCR was performed to detect SARS-CoV-2 genomic RNA using standard RT-qPCR workflows. Viral RNA levels in the supernatant were used as a measure of extracellular viral output from infected cultures. Relative viral RNA abundance across Anx5 concentrations was used to generate dose–response curves. IC₅₀ values for reduction of extracellular viral RNA were calculated by nonlinear regression analysis in GraphPad Prism (version 4.0) software (GraphPad Software, LLC; https://www.graphpad.com). These measurements were interpreted as reflecting changes in overall virus production and/or spread within the infected cell population rather than intracellular replication kinetics. All SARS-CoV-2 infections, cell viability and qPCR assays were carried out in the ImPaKT Facility Containment Level 3 laboratory (Western University, Ontario, Canada).

### SARS-CoV-2 Spike pseudovirus entry assay

Lentiviral particles expressing SARS-CoV-2 Spike (USA-WA1/2020) were produced as described by Crawford et al.^42^. Plasmids for pseudovirus production were obtained through BEI Resources, NIAID, NIH: SARS-Related Coronavirus 2, Wuhan-Hu-1 Spike-Pseudotyped Lentiviral Kit, NR-52,948. Briefly, 293T cells (ATCC, CRL-3216) were seeded at 4.8 × 10^6^ cells/ml in a 10 cm culture dish and maintained in DMEM supplemented with 100 U/ml penicillin, 100 µg/ml streptomycin, and 10% v/v FBS (Wisent Bioproducts) for 24 h, and the culture medium was replaced with 6 ml complete DMEM. Plasmid mix was prepared by diluting 1.32 µg of each helper plasmid (Tat, Rev, and Gag/Pol; BEI Resources, cat. no. NR-525-18, NR-52519, and NR-52517, respectively), 6 µg of the lentiviral backbone plasmid (BEI Resources, cat. no. NR-52516), and 2.04 µg of Spike expression plasmid pTwist-SARS-CoV-2 Δ18 (Addgene cat. no. 164436, a gift from Alejandro Balazs) in 500 µl DMEM. Transfection reagent mix was prepared by diluting 60 µl PolyJet transfection reagent (SignaGen Laboratories, cat. no. SL100688) in 500 µl DMEM. The transfection reagent mix was immediately added to the plasmid mix, incubated at room temperature for 10 min, and added to cells dropwise. The cells were incubated in a humidified cell culture incubator with 5% CO_2_ at 37 °C for 18 h, and the medium was replaced with 10 ml complete DMEM, and cultured for 60 h. Post-transfection, the pseudovirus-containing supernatant was removed from the cells, sterile-filtered (0.45 μm) and stored at -80 °C.

For pseudovirus entry assay, 293T-ACE2 cells in complete DMEM were seeded at 4 × 10^4^ cells per well in a 96-well plate coated with poly-L-lysine (Sigma-Aldrich, cat. no. P4707) and cultured for 24 h. The culture medium was replaced with 60 µl DMEM containing Anx5 at varying concentrations (0–200 µg/ml). SARS-CoV-2 Spike pseudovirus diluted (1:2) in DMEM was also pre-treated with Anx5 at corresponding concentrations. The cells and pseudoviruses with Anx5 were incubated for 1 h at 37 °C, 5% CO_2_ incubator. Pseudovirus/Anx5 mix (60 µl) was added to each cell/Anx5 mix at the corresponding Anx5 concentration and incubated for 1 h at 37 °C. The treated cells were washed with phosphate-buffered saline (PBS, Wisent Bioproducts, cat. no. 311-425-CL), and cultured in low serum (2% v/v FBS) DMEM for 48 h. The medium was replaced with 50 µl PBS containing calcium and magnesium (Wisent Bioproducts, cat. no. 311-420-CL), and 50 µl britelite plus luciferase reporter reagent (PerkinElmer, cat. no. 6066761) was added to the wells. 100 µl mixture from each well was transferred to a 96-well opaque black plate, and luminescence was read on a Cytation 5 plate reader with Gen5 Microplate Reader Software (version 3.02) software (Agilent BioTek; https://www.agilent.com).

### Time-of-addition assay for Anx5 during SARS-CoV-2 infection

Time-of-addition experiments were performed to assess the stage-specific effects of Anx5 on SARS-CoV-2 infection, as adapted from established antiviral assay frameworks. Human 293T cells stably expressing ACE2 (293T-ACE2) were seeded in 12-well plates at a density of 5–7 × 10⁵ cells per well in complete DMEM (DMEM supplemented with 10% fetal bovine serum and 1% penicillin–streptomycin) and incubated overnight at 37 °C with 5% CO₂ to achieve ~ 80–90% confluency at the time of infection. Cells were infected with SARS-CoV-2 (Wuhan strain, WA-1 P2) at a MOI of 0.01. Prior to infection, culture media were removed and replaced with virus inoculum diluted in complete DMEM. To synchronize viral entry, plates were subjected to spinoculation at 1,000 × g for 1 h at 4 °C, allowing viral binding without entry. Following centrifugation, the inoculum was removed, and cells were replenished with 700 µL of pre-warmed complete DMEM before transfer to 37 °C to initiate infection (defined as t = 0 h). Wild-type Anx5 was added to designated wells at a final concentration of 5 µg/mL at defined timepoints post-infection (0, 1, 2, 4, 6, 8, 10, 12, and 18 h). Anx5 was prepared in 1×PBS and added directly to culture media without media replacement to minimize perturbation of infection conditions. Each well received Anx5 only once and was incubated until the final 24 h endpoint. Control conditions included cells treated with a Ca^2+^-binding-deficient Anx5 mutant (E72Q, D144N, E228Q, D303N) at the same concentration. At 24 h post-infection, culture supernatants were collected to quantify extracellular viral RNA. Briefly, cells and supernatants were harvested, and samples were centrifuged at 500 × g to pellet cellular debris. Supernatants were transferred to fresh tubes and stored at -80 °C until analysis. Viral RNA was extracted using magnetic bead-based purification (MagMAX Viral RNA Isolation Kit, Thermo Fisher Scientific) and quantified by TaqMan-based quantitative PCR targeting the SARS-CoV-2 N gene. All qPCR measurements were normalized to the matched mutant Anx5 control within each biological replicate and expressed as fold change (wild-type/mutant).

### Binding model fits and statistical analyses

We used a one-site binding, ligand depletion model in all in vitro binding experiments to ensure accurate K_D_ determinations in regimes where total protein concentration [P]_t_ is comparable to K_D_. In this established thermodynamic model, the concentration of ligand bound (PL) is determined using a quadratic equation that accounts for the depletion of free ligand during the titration:

$$\:\:\left[PL\right]=\:\frac{\left({\left[P\right]}_{t}+{\left[L\right]}_{t}+{K}_{D}\right)-\:\sqrt{{\left({\left[P\right]}_{t}+{\left[L\right]}_{t}+{K}_{D}\right)}^{2}-4{\left[P\right]}_{t}{\left[L\right]}_{t}}\:}{2}$$

where, [P]_t_ is the total protein concentration (constant during the titration), [L]_t_ is the total ligand concentration (changing during the titration), K_D_ is the equilibrium dissociation constant.

For competitive binding assays, statistical comparison of the best-fit K_D_ values was performed using an extra sum-of-squares *F*-test. One-way ANOVA followed by Bonferroni or Dunnett’s multiple comparisons tests were applied for comparisons of steady-state fluorescence wavelength at maximal intensity, pseudovirus entry, and cell viability. For the time-of-addition experiment, statistical analyses were conducted on fold-change values using two-sided one-sample t-tests relative to the mutant-normalized baseline (fold-change = 1.0), with Holm correction applied for multiple comparisons. For grouped analyses, early (1–4 h) and late (6–18 h) timepoints were compared using Welch’s two-sided t-test, with additional confirmation using a Mann-Whitney U test.

## Supplementary Information

Below is the link to the electronic supplementary material.

Supplementary Material 1

Supplementary Material 2

Supplementary Material 3

Supplementary Material 4

Supplementary Material 5

## Data Availability

The Spike-RBD crystal structure coordinates were previously published^9^ and are accessible from the RCSB Protein Data Bank using accession code 6LZG.pdb ( https:/www.rcsb.org/structure/6LZG ). The ^15^N-Spike-RBD amide chemical shift assignments were previously published^37^ and are available at the Biological Magnetic Resonance Bank using accession code 50809 ( https:/doi.org/10.13018/BMR50809 ). All other data presented in this manuscript are available upon request to P.B.S. ( pstatho@uwo.ca ).

## Abbreviations

COVID-19: Coronavirus disease 2019
SARS-CoV-2: Severe acute respiratory syndrome coronavirus 2
ACE2: Angiotensin-converting enzyme 2
ARDS: Acute respiratory distress syndrome
Anx5: Annexin A5
RBD: Receptor binding domain
