# Supplementary material for: Dual engagement of Spike and ACE2 by annexin A5 contributes to pleiotropic SARS-CoV-2 inhibition
Source: Sci Rep. 2026 Jun 20;16:28261. doi: 10.1038/s41598-026-58451-9 (PMC13558681; doi:10.1038/s41598-026-58451-9)
Supplement: Supplementary file 1 [file 41598_2026_58451_MOESM4_ESM.docx]

**Table S1.** Summary of binding affinities measured using steady-state fluorescence (fl.), microscale thermophoresis (MST) and solution NMR spectroscopy.

| **protein** | **titrant** | **experiment** | **K_D_ (μM)^a^** | **K_D, err_ (μM)^b^** |
| --- | --- | --- | --- | --- |
| Anx5-fluorescein | Spike-RBD | steady-state  fl. intensity^c^ | 0.025 | 0.009 |
| Anx5-fluorescein | ACE2 | steady-state  fl. intensity^c^ | 0.023 | 0.010 |
| Anx5-fluorescein | truncACE2 | steady-state  fl. intensity^c^ | 0.024 | 0.015 |
| Anx5-fluorescein | Spike-RBD | steady-state  fl. λ_max_^d^ | 0.031 | 0.012 |
| Anx5-fluorescein | ACE2 | steady-state  fl. λ_max_^d^ | 0.054 | 0.048 |
| ACE2-RED-NHS | Spike-RBD | MST | 0.020 | 0.010 |
| ACE2-RED-NHS | Anx5 | MST | 8.8 | 0.4 |
| Anx5-fluorescein | ACE2 | MST | 0.30 | 0.20 |
| Anx5-fluorescein | Spike-RBD | MST | 10.0 | 8.0 |
| ^15^N-Spike-RBD | Anx5 | NMR | 0.4^e^ | 1.6 |
| ^15^N-Anx5 | Spike-RBD | NMR | 49^e^ | 24 |
| ^15^N-Anx5 | truncACE2 | NMR | 141^e^ | 86 |
| ^15^N-Anx5 | Spike-RBD | NMR | 6.5^f^ | 8.5 |
| ^15^N-Anx5 | truncACE2 | NMR | 3.4^f^ | 13 |

^a^Equilibrium dissociation constants (K_D_) were estimated using a one site binding model that takes into account protein concentration.

^b^Errors are standard errors of the fitted lines to the data.

^c^Binding isotherms were constructed from changes in fluorescence intensity (fl. intensity).

^d^Binding isotherms were constructed from changes in the wavelength at maximum fluorescence intensity (fl. λ_max_).

^e^K_D_s are global simultaneous fits to the CSPs observed for 4-6 peaks.

^f^K_D_s are global simultaneous fits to the CSPs observed for the 3 tightest peaks.
