# Supplementary material for: Dual engagement of Spike and ACE2 by annexin A5 contributes to pleiotropic SARS-CoV-2 inhibition
Source: Sci Rep. 2026 Jun 20;16:28261. doi: 10.1038/s41598-026-58451-9 (PMC13558681; doi:10.1038/s41598-026-58451-9)
Supplement: Supplementary file 2 [file 41598_2026_58451_MOESM5_ESM.docx]

**Figure S1.** Positive ionization electrospray mass spectrum of recombinantly expressed and purified Spike-RBD. Approximately 5 μM Spike-RBD was dialyzed into 10 mM ammonium bicarbonate buffer supplemented with 2 mM DTT prior to the denaturing LC-MS (see Methods). The theoretical molecular weight of 6×His-Spike-RBD is 27,419.9 Da (average isotopic atom masses and excluding the start-Met). The recombinant protein contains 9 Cys. The measured mass of 27,412.6 Da is 7.3 Da less than the theoretical molecular weight, which can be attributed to the formation of 4 disulfides that decrease the molecular weight by 8 Da. Note that the crystal structure of Spike-RBD also shows 4 intramolecular disulfides (6LZG.pdb).

**Figure S2.** SEC-MALS analysis of Anx5 interaction with Spike-RBD and ACE2. **(A)** SEC-MALS elution profiles of Spike-RBD alone (3 mg/mL, red line), Anx5 alone (1 mg/mL, blue line) and Spike-RBD mixed with Anx5 (3:1 mg/mL, dashed black line). MALS-determined molecular weights calculated over the Spike-RBD, Anx5 and mixed elution peaks are shown as red, blue and black circles, respectively. Coomassie blue-stained SDS-PAGE gels of the **(B)** Spike-RBD alone, **(C)** Anx5 alone, and **(D)** Spike-RBD mixed with Anx5 elution fractions. **(E)** SEC-MALS elution profiles of ACE2 alone (0.7 mg/mL, red line), Anx5 alone (1 mg/mL, blue line) and ACE2 mixed with Anx5 (0.7:1 mg/mL, dashed black line). MALS-determined molecular weights calculated over the ACE2, Anx5 and mixed elution peaks are shown as red, blue and black circles, respectively. Coomassie blue-stained SDS-PAGE gels of the **(F)** ACE2 alone, **(G)** Anx5 alone, and **(H)** ACE2 mixed with Anx5 elution fractions. **(I)** SEC-MALS elution profiles of truncACE2 alone (1.5 mg/mL, red line), Anx5 alone (1 mg/mL, blue line) and truncACE2 mixed with Anx5 (1.5:1 mg/mL, dashed black line). MALS-determined molecular weights calculated over the truncACE2, Anx5 and mixed elution peaks are shown as red, blue and black circles, respectively. Coomassie blue-stained SDS-PAGE gels of the **(J)** truncACE2 alone, **(K)** Anx5 alone, and **(L)** ACE2 mixed with ACE2 elution fractions. All experiments were performed in 20 mM Na_2_HPO_4_ (pH=7.4) at ~10°C. Data are representative of n=1-3 experiments.

**Figure S3.** Comparison of the ACE2:Spike-RBD and Anx5:Spike-RBD interfaces. **(A)** Crystal structure of the human ACE2 extracellular domain (transparent maroon cartoon) in complex with the SARS-CoV-2 Spike-RBD (white surface) (6LZG.pdb) ^9^. Spike-RBD residues in the ACE2:Spike-RBD interface were extracted from the PDBSum^59^ analyses and are coloured blue. **(B)** SARS-CoV-2 Spike-RBD surface representation of the Anx5-induced broadening. Loss in amide peak intensity is coloured from white (minimum change in I/I_0_) to orange (medium change in I/I_0_) to red (maximum change in I/I_0_), where I and I_0_, are the Spike-RBD (~150 μM) amide peak intensities in the absence and presence of ~500 μM Anx5. Spike-RBD assignments were transferred from BMRB entry 50809 ^40^. In *A* and *B*, the images from left to right across the page are rotated 90° rightward relative to one another.

**Reference**

59	Laskowski, R. A., Jablonska, J., Pravda, L., Varekova, R. S. & Thornton, J. M. PDBsum: Structural summaries of PDB entries. *Protein Sci* **27**, 129-134, doi:10.1002/pro.3289 (2018).
